# New Insights into MAI Additives in 2D‐Assisted 3D Controlled Crystallization Toward High‐Quality *α*‐Phase FAPbI_3_ Perovskites

**DOI:** 10.1002/advs.202402065

**Published:** 2024-08-06

**Authors:** Tao Liu, Meichen Hou, Wending Hao, Shitong Du, Wenbin Yang, Yihui Yuan, Ning Wang

**Affiliations:** ^1^ State Key Laboratory of Marine Resource Utilization in South China Sea Hainan University Haikou 570228 P. R. China

**Keywords:** 2D template, MAI, perovskite solar cells, preferred crystallographic orientation, *α*‐FAPbI_3_

## Abstract

The highly oriented 2D perovskite templates of n = 1 have typically been created to attain controllable and oriented crystallization of 3D α‐phase formamidinium lead triiodide (*α*‐FAPbI_3_) perovskites. However, the role of methylammonium iodide (MAI), a widely used *α*‐FAPbI_3_ phase stabilizer, in regulating the growth dynamics of 2D/3D perovskites is generally ignored. Herein, Ruddlesden–Popper type n = 1 2D octylammonium lead iodide (OA_2_PbI_4_) perovskites are added into FAPbI_3_ precursor solution. The template of n = 2 (OA_2_MAPb_2_I_7_), which is spontaneously constructed by the mixture of n = 1 2D and methylammonium chloride (MACl), acts as a skeleton to template the epitaxial growth of *α*‐FAPbI_3_. However, the volatilization of MACl inevitably causes damage to the 2D structure during thermal annealing. This study reveals that small amounts of less volatile MAI additive enables the creation of stable 2D template, leading to more controlled vertical orientation crystallization. Consequently, the high‐quality mixed‐dimensional perovskite film delivers a high efficiency of 24.19% together with improved intrinsic stability. This work provides an in‐depth understanding of 2D‐assisted controlled epitaxial growth of *α*‐FAPbI_3_.

## Graphical abstract

MAI dopants are commonly introduced in 2D/3D hybrid perovskites to stabilize the crystal structure of *α*‐FAPbI_3_, while the role of MAI in regulating the growth dynamics of *α*‐FAPbI_3_ is generally ignored. It reveals that small amounts of MAI make the 2D template more stable, leading to controlled crystallization and a strong degree of preferential orientation for α‐FAPbI_3_ perovskites.

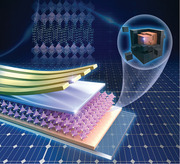

## 1 Introduction

Owing to the overwhelming optoelectronic properties and the cost‐effective, solution‐ processable preparation, hybrid organic–inorganic perovskites have emerged as foremost contenders for future photovoltaic technology.^[^ ^1^ ^]^ Within a short period of time, perovskite solar cells (PSCs) based on 3D perovskites have experienced surge development and the certified record power conversion efficiency (PCE) has increased to 26.1% for single‐junction PSCs.^[^ ^2^ ^]^ Among lead halide perovskites, *α*‐phase formamidinium (FA)‐based lead triiodide (*α*‐FAPbI_3_) has emerged as the strongest candidate due to its small bandgap [*E*_g_ ≈ 1.48 eV in thin films], approaching the Schockley–Queisser limit.^[^ ^3^ ^]^ *α*‐FAPbI_3_ also affords improved thermal stability relative to methylammonium lead triiodide (MAPbI_3_) due to its elevated decomposition temperature.^[^ ^3^ ^]^ Unfortunately, the trigonal black *α*‐FAPbI_3_ readily transforms into the photoinactive yellow δ‐phase with hexagonal symmetry under ambient conditions at room temperature since the large size of FA^+^ induces lattice distortion, which undermines the photovoltaic efficiency and long‐term stability of the PSCs.^[^ ^4^ ^]^ A great deal of efforts to inhibit this phase transformation of FAPbI_3_ have focused on compositional engineering by partially replacing FA/Iodide (I) with mixing alternative cations, anions or both to form hybrid perovskites, such as MA, cesium and rubidium cations, chlorine and bromine anions.^[^ ^5^ ^]^ However, tuning of compositions will inevitably widen its bandgap and possible local phase segregation caused by non‐native ion migration.^[^ ^6^ ^]^ Therefore, instead of using a substitution strategy, functional volatile alkyl ammonium chloride (RACl) (e.g., methylammonium chloride (MACl),^[^ ^7^ ^]^ propylamine chloride (PACl),^[^ ^8^ ^]^ methylenediammonium dichloride (MDACl_2_)^[^ ^9^ ^]^) dopants/additives, which can bind to [PbI_6_]^4−^ octahedron to form the intermediate phase, have been proven essential in low‐temperature formation, grain growth and preferential orientation of *α*‐FAPbI_3_. MACl is currently one of the most prevalent dopants for preparing high‐performance *α*‐FAPbI_3_ PSCs. On the one hand, MACl additive can quickly induce MA‐rich nuclei to form pure *α*‐phase FAPbI_3_ and dramatically reduce phase‐transition temperatures, thereby resulting in phase stability.^[^ ^10^, ^11^ ^]^ On the other hand, volatile MACl provides a unique effect on promoting the growth of secondary crystallization during annealing.^[^ ^10^, ^12^ ^]^ It is well known that the crystallinity is crucial for the stability of the perovskites, because the main degradation process starts from defects near the grain boundaries (GBs).^[^ ^13^ ^]^ The high crystallinity and large grain size of the *α*‐FAPbI_3_ perovskite films will contribute to their greater stability and improved device performance.^[^ ^14^, ^15^ ^]^ However, the inconsistency of the local orientation of the crystals will inevitably lead to stacking defects in FAPbI_3_ perovskites with 35 mol% additional MACl,^[^ ^16^ ^]^ hereafter denoted “Control”. Meanwhile, the large local residual stress also leads to an increase in the internal point defects of perovskite films and promotes the migration of halide ions, which is manifested as an accelerated perovskite degradation process.^[^ ^17^ ^]^

Attaining an oriented crystallization remains a significant challenge because of the lack of highly ordered nuclei seed in the as‐casted semiwet film for initiating the crystallization step.^[^ ^18^, ^19^ ^]^ Thus far, RA^+^ with longer lengths or larger ionic radii than that of FA^+^ and MA^+^ have been mainly used in forming 1D or 2D lead halide perovskites, which are incorporated within a 3D perovskite network for defect passivation of GBs and surfaces.^[^ ^20^ ^]^ More importantly, once a highly matched heterointerface is formed between the highly oriented 2D perovskite and cubic *α*‐FAPbI_3_, 2D template can guide the “heteroepitaxial growth” of *α*‐FAPbI_3_,^[^ ^18^ ^]^ which lowers the crystallization energy and induces a high degree of crystal orientation and a low residual stress.^[^ ^21^ ^]^ Moreover, such an epitaxial growth strategy triggers the direct conversion of PbI_2_ to *α*‐FAPbI_3_ without the involvement of the yellow δ‐phase.^[^ ^18^ ^]^ Consequently, the improved crystal quality and film uniformity substantially increase charge transporting characteristics, and suppress nonradiative recombination losses.^[^ ^22^, ^23^ ^]^ Dimensional engineering has become an effective strategy to improve both efficiency and stability. Generally, 2D templates of n = 1 have been widely used to regulate the ordered growth of *α*‐FAPbI_3_.^[^ ^24^, ^25^, ^26^ ^]^ As previous mentioned, small amounts of MAI dopants are commonly introduced in 2D/3D hybrid systems to stabilize the crystal structure of *α*‐FAPbI_3_, whereas the role of MAI in the nucleation and crystallization of *α*‐FAPbI_3_ is generally ignored.^[^ ^27^, ^28^, ^29^ ^]^ Herein, we provide key missing information on how MAI regulates the growth dynamics of 2D/3D perovskite films for the desired facet orientation and stacking mode.

Herein, we designed a 2D/3D heterostructure based on FAPbI_3_ 3D perovskites and Ruddlesden–Popper (RP) type n = 1 2D perovskites (OA_2_PbI_4_, OA is octylammonium). We gained insight into the formation mechanism of 2D/3D perovskites using in situ optical diagnostics during spin coating and subsequent thermal annealing. When OA_2_PbI_4_ single crystals (SCs) were added into the precursor solution of Control perovskites, the spontaneous transition from n = 1 to n = 2 (OA_2_MAPb_2_I_7_) allowed the preferential formation of 2D template of n = 2, which enabled the direct epitaxial growth of *α*‐FAPbI_3_ (hereafter denoted as “TD”). However, 2D templates were partially destroyed due to the volatilization of MACl during annealing at 150 °C. We reveal that small amounts of MAI additives added into TD precursor solution can stabilize 2D template structure, which results in more controlled crystallization along the (100) facet. As a result, the desired crystallization and stacking mode can be achieved for *α*‐FAPbI_3_ perovskites, which is referred to as “MTD”. Our study highlights the critical role of MAI additive on the controllable and oriented crystallization of *α*‐FAPbI_3_.

## 2 Results and Discussion

### 2.1 Structure, Morphology and Optoelectronic Properties

**Figure 1:**
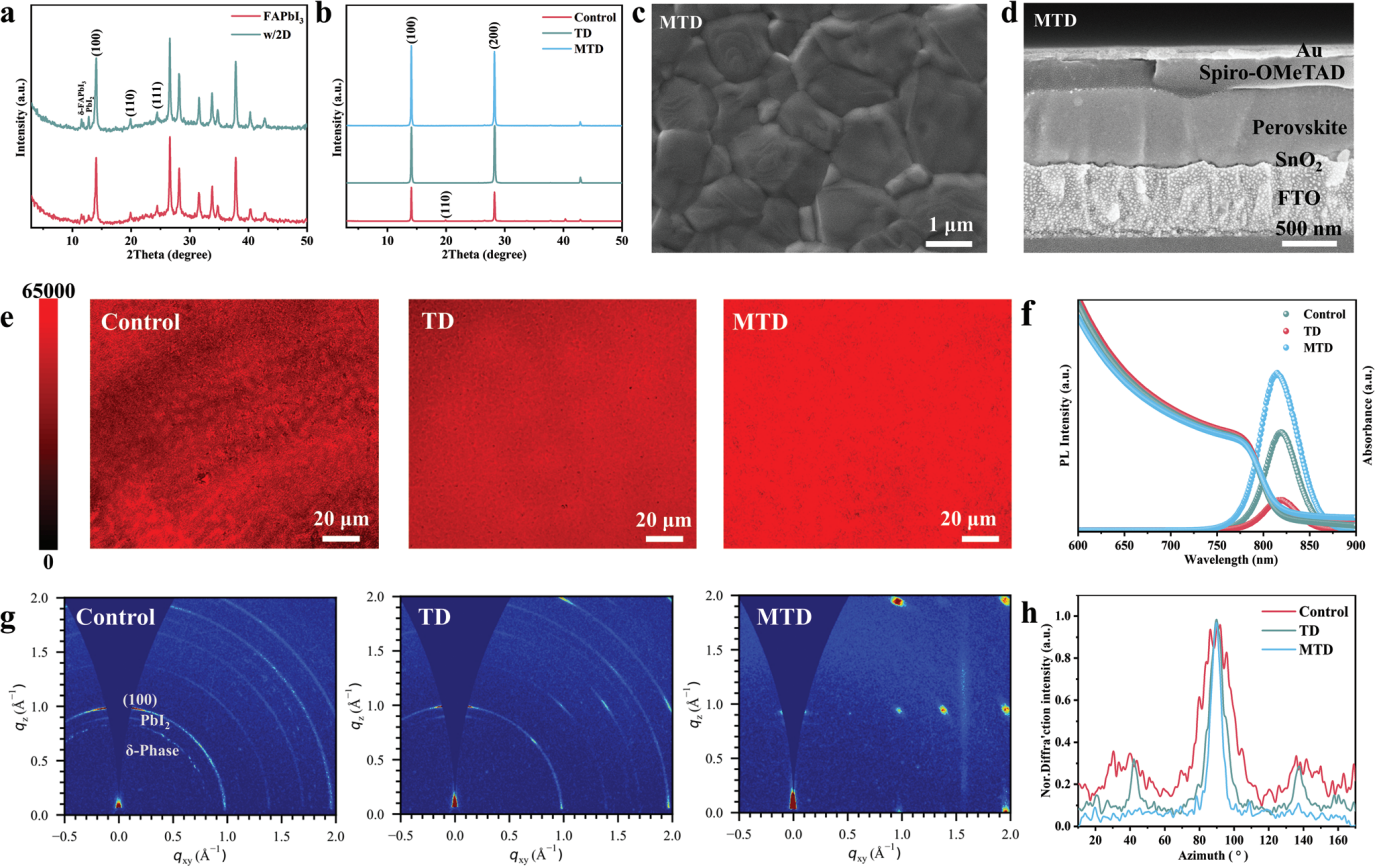
Structure, optical properties and facet orientation of Control, TD, MTD perovskite films. a) XRD patterns of pristine FAPbI_3_ and FAPbI_3_ with 2D RP phase OA_2_PbI_4_ (denoted as w/2D). b) XRD patterns of Control, TD, MTD perovskite films. c) Top‐view SEM image of MTD perovskite film. d) Cross‐section SEM image of the device assembled with MTD perovskite film. e) PL mappings of Control, TD, MTD perovskite films using CLSM. f) UV–vis absorption spectra and steady‐state PL spectra of Control, TD, MTD perovskite films. g) GIWAXS characterizations of Control, TD, MTD perovskite films. h) Azimuth of GIWAXS plots azimuthally along the ring at *q_xy_* = 10 nm^−1^.

Figure S1 (Supporting Information) shows the X‐ray diffraction (XRD) pattern of 2D OA_2_PbI_4_ SCs, consistent with the simulated result by density functional theory (DFT) calculation. Perovskite thin films were prepared by spin‐coating method and then annealed at 150 °C (see more details in experimental procedures). When the as‐prepared 2D SCs were added into the precursor solution of the compositional‐pure FAPbI_3_ (without addition of MACl), the resulting perovskite film exhibited identical multi‐peak positions with the compositional‐pure FAPbI_3_ (**Figure** **1**a), indicating that 2D SCs did not change the crystalline packing model of 3D FAPbI_3_ perovskites.^[^ ^30^ ^]^ As shown in Figure 1b, as expected, the addition of 35 mol % additional MACl improved the crystallinity of *α*‐phase perovskite, exhibiting (100), (110), and (200) peaks located at 2 theta = 14.0°, 19.9°, and 28.0°, respectively.^[^ ^31^ ^]^ Notably, the TD film shows enhanced peak intensity of (100) facet with negligible (110) peak (Figure S2, Supporting Information), suggesting that the existence of MACl allows for 2D templates‐assisted controlled nucleation/growth of 3D perovskites. This finding is further verified by the improved crystallinity of 2D SCs incorporated FAPbI_3_ perovskites, Figure S3 (Supporting Information). Importantly, MTD shows the highest peak intensities with an increase of almost three orders of magnitude in the number of counts for (100) and (200) planes compared to the Control film. It is known that the highly preferred crystallographic orientation of the (100) facets can substantially increase charge transporting and suppress nonradiative recombination losses.^[^ ^32^ ^]^ Herein, the molar ratio of 2D to MAI is set to 1:1, and the optimized content of MAI (or 2D) is 0.2 mol% relative to FAI, Figure S4 (Supporting Information). Unless stated otherwise, all the characterizations were performed on the optimized MTD films. Compared to the Control film, TD and MTD films exhibit the elimination of pinholes and defects in the polycrystalline perovskites with more compact grains, Figure 1c and Figure S5 (Supporting Information). The cross‐sectional scanning electron microscope (SEM) image of MTD in Figure 1d illustrates the monolithic grains from the top to the bottom without obvious voids. The well‐stacked perpendicular perovskite grains can transport carriers without traversing GBs, thereby facilitating the carrier migration and suppressing the nonradiative recombination.^[^ ^33^ ^]^ In striking contrast, there are obvious pinholes and highly unordered perovskite grains in the Control sample, Figure S6a (Supporting Information). TD film shows closely packed grains, but not a monolayer across the thin film, Figure S6b (Supporting Information). Atomic force microscope (AFM) images in Figure S7 (Supporting Information) illustrate a smoother TD and MTD film with a decreased root‐mean‐square (RMS) roughness of 27.0 and 24.8 nm, respectively, compared to that of the Control film (68.7 nm), implying a better interfacial contact between the perovskite film and hole transporting layer (HTL), as evidenced by steady‐state PL spectra of perovskite/HTL samples (Figure S8, Supporting Information).^[^ ^34^ ^]^ To gain insights into the film homogeneity and defect distribution on a micrometer scale, Figure 1e shows the photoluminescence (PL) mapping of perovskite films monitored at 800 nm using confocal scanning laser microscopy (CSLM). Obviously, the added 2D SCs result in overall enhanced PL intensity and improved uniformity. Compared with TD sample, the relatively high PL intensity of *α*‐FAPbI_3_ results in MTD one created a striking contrast between perovskite grains and GBs, thereby leading to the appearance of micro cluster structure. Figure 1f shows the ultraviolet–visible (UV–vis) absorption and steady‐state PL spectra of the *α*‐FAPbI_3_ perovskite films. The absorption threshold and PL peak position were identical for all films, while MTD film delivered a higher PL intensity than that of TD film together with a slight blue shift, confirming the high‐quality perovskite film due to the synergic effect of 2D phases and MAI, Figure 1f. We further compared the orientation of the diffracting crystallites by grazing‐incidence wide‐angle X‐ray diffraction (GIWAXD) analysis, Figure 1g. The facet stacking mode is essential for carrier transport in perovskite films.^[^ ^35^ ^]^ The Control film exhibited the diffraction ring at *q_xy_* = 1.0 Å^−1^ assigned to (100) facet, and *q* is the scattering vector in reciprocal space.^[^ ^36^ ^]^ The strong Debye–Scherrer ring with an isotopic intensity distribution in the Control film indicates that the grain orientation is completely random, which suppress vertical transport of carriers.^[^ ^37^ ^]^ Remarkably, MTD film shows the sharp Bragg spot of (100) plane along the out‐of‐plane (*q_z_*) direction, indicating well‐aligned *α*‐FAPbI_3_ perovskite.^[^ ^38^ ^]^ A relatively weak isotopic intensity distribution can be observed in the TD film. We integrated the GIWAXS pattern azimuthally over the diffraction ring at *q_xy_* = 1.0 Å^−1^, Figure 1h. For the Control film, the diffraction ring exhibited three broad peaks at ≈35°, ≈90°, and ≈145°. In comparison, the width of the three peaks become much narrower for TD film, indicating improved crystallinity and grain orientation.^[^ ^39^ ^]^ Furthermore, the peak located at the 90° azimuth angle appeared the minimum full width at half maximum (FWHM) value in the MTD film accompanied by the disappearance of other two peaks at 35° and 145°. This indicates that most (100) facets tend to stack parallel to the substrate.^[^ ^40^ ^]^ The highly ordered and aligned perovskite grains across the thin film improve the vertical transport of carriers and the corresponding photovoltaic performance of PSCs.^[^ ^41^ ^]^ Overall, MAI additives play a major role in regulating 2D templates‐induced controlled growth of the 3D perovskite during the crystallization process.

### 2.2 In Situ Investigation into 2D Template‐Induced Crystallization Kinetics

**Figure 2:**
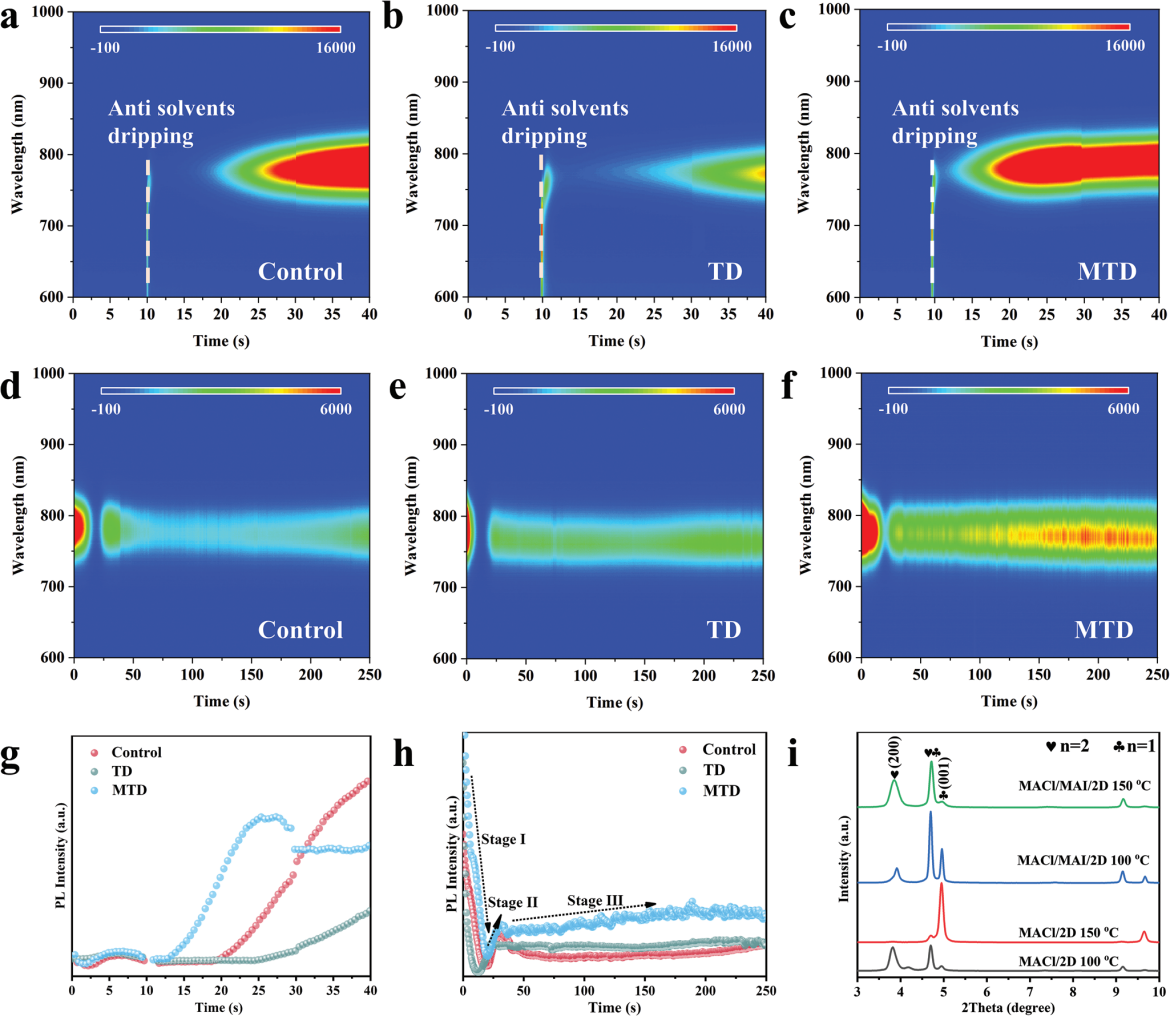
In situ PL measurements for the crystallization kinetics of FAPbI_3_ perovskite films. a‐f) In situ PL spectra of Control, TD and MTD perovskite films during spin coating (a, b, c) and annealing (d, e, f). g) Extracted PL peak intensity during spin coating and h) annealing. i) XRD patterns of MACl/2D and MACl/MAI/2D films after annealing at 100 and 150 °C. The precursor solution of MACl/2D was prepared by dissolving MACl and 2D SCs into a mixed solution of DMF and DMSO.

In situ PL spectroscopy was performed for real‐time monitoring of the crystallization process during spin coating and subsequent thermal annealing of Control, TD, and MTD films, **Figure** **2**a‐f. The corresponding PL peak positions and intensities were extracted and plotted in Figure 2g,h, respectively. To detect 2D transition, the added concentration of 2D SCs was increased from 0.2 to 2 mol%. As shown in Figure S9 (Supporting Information), we observed the formation of OA_2_MAPb_2_I_7_ (n = 2) with a PL peak centered at ≈615 nm. The corresponding steady‐state PL spectra of n = 1 and n = 2 2D SCs are displayed in Figure S10 (Supporting Information). Figure 2a‐c presents the time‐dependent change in PL intensity during spin coating. The *α*‐phase perovskite crystallizes at the point when anti‐solvent dripping was applied at ca. 10 s during spin coating. It is widely acknowledged that highly oriented 2D act as templates for regulating the growth dynamics of *α*‐FAPbI_3_ and making its crystallization stride directly over the nucleation.^[^ ^25^, ^42^, ^43^ ^]^ Hence, the increased PL intensity can be partially ascribed to the direct growth of the 3D perovskite phase. It was obvious that TD film presented progressively slower crystallization process than the Control one (Figure 2g), implying that the addition of 2D perovskites slowed down the crystallization kinetics during spin coating. This finding may be ascribed to the fact that MACl was involved in nucleation and growth of n = 2 2D perovskites, which created the direct competitive with the formation of MA‐rich nuclei and hence enabled the crystallization of *α*‐FAPbI_3_ to slow down. In sharp contrast, the PL intensity of MTD exhibited a sharp increase upon antisolvent dripping, Figure 2g. One possible explanation is that MAI participates in preferential growth of n = 2 2D perovskites, which have no chance of competing against MACl intermediate nucleus. Subsequently, such a 2D template guided the free components to form 3D *α*‐phase perovskite with the desired facet orientation and stacking mode.^[^ ^26^ ^]^ Figure 2d‐f shows the time‐dependent change in PL intensity during annealing process. The PL intensity in Figure 2h exhibited a sharp drop during the first 10–20 s (Stage Ι), which was caused by the dissolution of the *α*‐phase perovskite on the surface when the solvent escaped from the bulk film as reported.^[^ ^28^ ^]^ Furthermore, the dissolution‐recrystallization equilibrium of the *α*‐phase perovskite resulted in a continuous increase of PL intensity until 25–30 s (Stage II). During stage III, the Control film showed a slight decrease in the PL intensity. MACl participated in the formation of randomly orientated MA‐rich nuclei, which resulted in the inconsistency of the local orientation of the *α*‐FAPbI_3_ crystals, leading to the formation of stacking defects.^[^ ^44^ ^]^ In contrast, MTD film exhibited a slight increase of PL intensity as thermal annealing was prolonged. This finding indicated that the 2D template acted as nucleation sites instead of MA‐rich nuclei, and successfully induced the epitaxial growth of *α*‐FAPbI_3_, thereby inhibiting the formation of stacking defects. Nevertheless, the PL peak of TD film exhibited negligible variation at this stage because the rapid release of volatile MACl resulted in collapse of partial templates of n = 2, as confirmed by XRD patterns, Figure 2i. We observed the formation of n = 2 2D structure for the mixture of MACl and n = 1 2D (MACl/2D) after annealing at 100 °C.^[^ ^45^ ^]^ However, the characteristic peak of n = 2 2D phase disappeared when the annealing temperature increased to 150 °C, resulting from the rapid release of volatile MACl.^[^ ^10^, ^46^ ^]^ Following the inclusion of MAI (MACl/MAI/2D), however, the diffraction signals of n = 2 2D become more prominent after annealing at 150 °C than that at 100 °C, suggesting more stable 2D phase, which contrasted with MACl/2D at 150 °C. It is worth noting that 2D template of n = 2 can be constructed by the mixture of n = 1 2D and MAI (instead of FAI) after annealing at 150 °C, Figure S11 (Supporting Information). For comparison, 2D SCs of n = 2 (OA_2_MAPb_2_I_7_) and n = 4 (OA_2_MA_3_Pb_4_I_13_) phases were also synthesized for in‐depth understanding of the crystallization dynamics of 2D‐assisted 3D growth. Compared with n = 1 2D case, the consistent behavior in the time‐dependent PL intensity can be observed for the perovskite films (without MAI) upon n = 2 or n = 4 SC treatment during spin coating and annealing, Figure S12 (Supporting Information). Furthermore, the similar peak positions and intensity in XRD patterns can be observed for the MTD film and 2D SCs of n = 2 treated perovskite film, Figure S13 (Supporting Information). Hence, it is reasonable to assume that MAI additive enables the construction of the stable 2D template of n = 2, which creates the right condition for a highly matched heterointerface with cubic 3D *α*‐FAPbI_3_.

**Figure 3:**
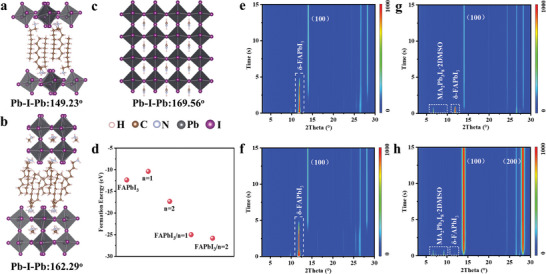
Crystalline kinetics mechanism analysis of FAPbI_3_ perovskite films. Structure and in‐plane distortion (Pb‐I‐Pb angle) for a) n = 1 2D (OA_2_PbI_4_), b) n = 2 2D (OA_2_MAPb_2_I_7_), and c) FAPbI_3_. d) The formation energy calculated with DFT method. FAPbI_3_/n = 1(n = 2) denotes the formation energy of FAPbI_3_ grown on n = 1(n = 2) 2D perovskite. e–h) Time‐dependent XRD patterns recorded for FAPbI_3_ perovskite films without MACl during annealing. (e) pristine FAPbI_3_, (f) FAPbI_3_ with n = 1 2D, (g) FAPbI_3_ with MAI, (h) FAPbI_3_ with MAI and n = 1 2D.

The in‐plane distortion (Pb‐I‐Pb angle) of the n = 1 and n = 2 2D perovskites were calculated using DFT for investigating matching degree of 2D/3D heterostructure. As shown in **Figure** **3**a,b and n = 1 phase demonstrated a smaller Pb‐I‐Pb angel of 149.23° than that of n = 2 phase (162.29°). A larger Pb‐I‐Pb angle indicates a better matched heterointerface between the (002) lattice plane of n = 2 2D and the (100) facet of 3D 𝛼‐FAPbI_3_ (Pb‐I‐Pb angle is 169.56°, Figure 3c).^[^ ^20^ ^]^ Based on the epitaxial growth theory, n = 2 2D acts as a skeleton to template the epitaxial growth of 𝛼‐FAPbI_3_ (FAPbI_3_/n = 2), which reduces the formation energy barrier from −12.4 eV (FAPbI_3_) to −25.8 eV (FAPbI_3_/n = 2) using DFT calculation (Figure 3d) according to the corresponding molecular structure models (Figure S14, Supporting Information). Since 2D phase of n = 2 has a lower formation energy than that of n = 1 2D, the spontaneous transition from n = 1 to n = 2 occurred when OA_2_PbI_4_ was added into perovskite precursor solution. In order to gain a deep understanding of the role of MAI in the crystallization kinetics, we also characterized time‐dependent XRD of FAPbI_3_ perovskite films in the absence of MACl during thermal annealing at 150 °C. It was observed that the pristine FAPbI_3_ perovskite film first formed a yellow δ‐phase, and then gradually converted to *α*‐phase over the annealing time (Figure 3e). Following the inclusion of n = 1 2D SCs (Figure 3f) or MAI (Figure 3g), the conversion from initial δ‐phase to *α*‐phase perovskite still occurred during the crystallization. Owing to the addition of MAI, MA_2_Pb_3_I_8_•2DMSO intermediate phase appeared together with δ‐phase perovskite,^[^ ^47^ ^]^ Figure 3g. By contrast, incorporating n = 1 2D and MAI almost eliminated δ‐FAPbI_3_ at the beginning of annealing, and the precursors directly crystalized to *α*‐FAPbI_3_, Figure 3h. The corresponding (100) diffraction intensity of black‐phase perovskite became stronger than other perovskites. This finding is in line with our previous conjecture that the template of n = 2 constructed by n = 1 2D and MAI preferentially forms in the initial stage of crystallization, and then induces the direct epitaxial growth of *α*‐phase FAPbI_3_ without the involvement of the yellow δ‐phase.

### 2.3 Schematic Illustration of 2D Template‐Induced Controlled Growth

**Figure 4:**
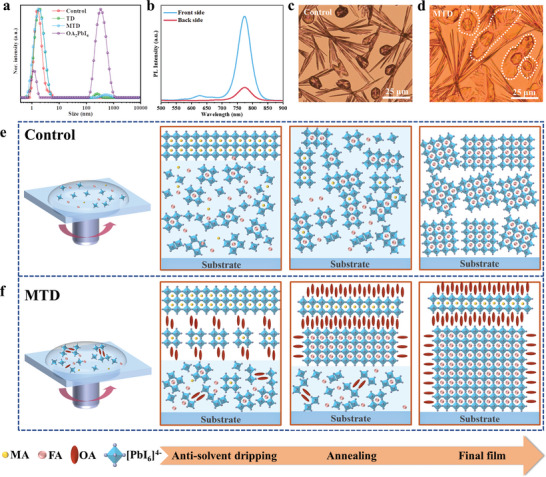
2D‐assisted 3D controlled crystal growth kinetics. a) Dynamic light scattering spectra of Control, TD, MTD perovskite precursor solution as well as n = 1 2D (OA_2_PbI_4_) SCs dissolved in DMF/DMSO solvents. b) Steady‐state PL spectra of MTD perovskite films on the glass excited at the front and back sides at the beginning of thermal annealing. Optical microscopy images of the wet c) Control and d) MTD perovskite films at 15 min without dripping the antisolvent. Schematic illustration of crystallization process of e) Control and f) MTD perovskite films.

The perovskite precursor solution was characterized by dynamic light scattering, **Figure** **4**a. The particle size in a Control solution was less than 10 nm. After inclusion of 0.2 mol% 2D SCs, the corresponding characteristic particle size of several hundred nanometers could be detected, confirming that 2D SCs still maintained the colloidal properties in the perovskite precursor solution.^[^ ^48^ ^]^ In addition, the particle size in MTD precursor solution was identical to that of TD solution, indicating that a small amount of MAI additive had little influence on the 2D colloidal particles. Figure 4b shows the PL spectra of MTD film grown on the glass at the beginning of thermal annealing to probe the preferential growth direction of the ordered FAPbI_3_ perovskite. The laser excitation on the perovskite surface and the glass surface is referred to as “Front side” and “Back side”, respectively. As shown in Figure 4b, Front side had a distinct PL peak at ≈625 nm assigned to n = 2 2D, while no 2D peak was observed on the Back side. It can be inferred that 2D templates of n = 2 are first formed on the air/liquid interface, and then immediately act as an epitaxial template for the direct conversion of PbI_2_ to *α*‐FAPbI_3_. 2D template induced crystallization will rapidly consume solute and significantly reduce solution concentration. This in turn inhibits the random nucleation of the remaining 3D perovskite,^[^ ^29^ ^]^ making the 2D template‐induced crystallization dominate. As a result, *α*‐FAPbI_3_ preferentially grew along the vertically ordered alignment, which contributed to the enhanced crystallinity and highly preferred crystallographic orientation of the (100) facets, resulting in efficient charge transfer and extraction. The optical microscopy images of the wet perovskite films without antisolvent dripping were recorded to visually present the crystallization process of *α*‐FAPbI_3_ perovskites. Figure S15 (Supporting Information) shows the time sequence of the crystallization kinetics of the Control perovskite film. The needles of MA_2_Pb_3_I_8_·2DMSO was first observed, and then the blocks of FAPbI_3_ appeared successively over time, Figure 4c and Figure S14 (Supporting Information). After the addition of MAI and 2D SCs in FAPbI_3_ precursor solution, n = 2 2D templates appeared prior to the emergency of MA_2_Pb_3_I_8_·2DMSO, Figure S16 (Supporting Information). As time goes on, the core‐shell structure can be clearly observed, confirming that the 2D template of n = 2 induced direct epitaxial growth of ordered *α*‐FAPbI_3_ without the participant of the yellow δ‐phase. The crystallization process of TD and MTD films exhibited negligible variation, Figure 4d and Figure S17 (Supporting Information). In addition, the crystallization process of the pristine MAPbI_3_ and FAPbI_3_ perovskites were also observed by optical microscopy simultaneously. As shown in Figures S18 and S19 (Supporting Information), the epitaxial growth did not contribute to the crystallization process due to the disappearance of core‐shell structure for wet MAPbI_3_ and FAPbI_3_ perovskite films. Furthermore, the core‐shell structure also did not appear in FAPbI_3_ perovskites added with n = 1 2D (Figure S20, Supporting Information) or MAI (Figure S21, Supporting Information). By contrast, we observed the core‐shell structure in the MTD film without MACl (Figure S22, Supporting Information), which further confirmed that n = 1 2D and MAI preferentially formed the template of n = 2 in the initial stage of crystallization. Specially, the disappearance of needles of MA_2_Pb_3_I_8_·2DMSO illustrated that MAI was not involved in the formation of intermediate phase. Figure S23 (Supporting Information) shows the digital photographs of the deposited perovskite films without dripping the antisolvent at room temperature. Notably, the Control and MTD films can turn black *α*‐phase after 30 min, while the yellow δ‐phase remained unchanged for FAPbI_3_ and FAPbI_3_/2D over time. Evidently, the phase transformation from yellow δ‐phase to black *α*‐phase was not observed for MTD film, in good agreement with our analysis results. As for FAPbI_3_/2D/MAI, the synergistic effect 2D SCs and MAI can help turn black *α*‐phase in the absence of MACl. Figure 4e,f shows schematic images comparing the crystallization processes of the Control and MTD films. The Control film exhibited preferential crystallization at the gas‐liquid interface during spin coating. Subsequently, the dissolution and recrystallization occurred when the entrapped solvent left the bulk film during thermal annealing.^[^ ^49^ ^]^ The homogeneous nucleation finally caused the volume collapse of perovskite crystals, resulting in stacking defects and local stress.^[^ ^50^ ^]^ As for MTD film, the addition of the 2D SCs did not change the dissolution of the perovskite in the initial stage of thermal annealing. The spontaneous transition from n = 1 to n = 2 occurred first on the gas–liquid interface during the dissolution and recrystallization, which can be supported by the distribution of MA^+^ in TOF‐SIMs of MTD‐based device (Figure S24, Supporting Information). Subsequently, 2D template of n = 2 provided a skeleton for the vertical orientation growth of 3D *α*‐phase without the involvement of the yellow δ‐phase. Specially, small amounts of MAI additives protected n = 2 2D structure from the volatilization of MACl during thermal annealing. The above‐mentioned growth pathways avoided the formation of voids and volume collapse when the trapped DMSO solvent escaped from the film, which is promising for crystallization regulation toward the achievement of high‐quality FAPbI_3_ perovskite film. The crystallization process of MTD and TD perovskites was roughly the same, whereas the partial destruction of 2D template resulted in the random nucleation and orientation. Even so, TD sample showed improved crystallographic orientation of the (100) facets compared with Control film, as evidenced by GIWAXS (Figure 1h).

### 2.4 Photovoltaic Performance

**Figure 5:**
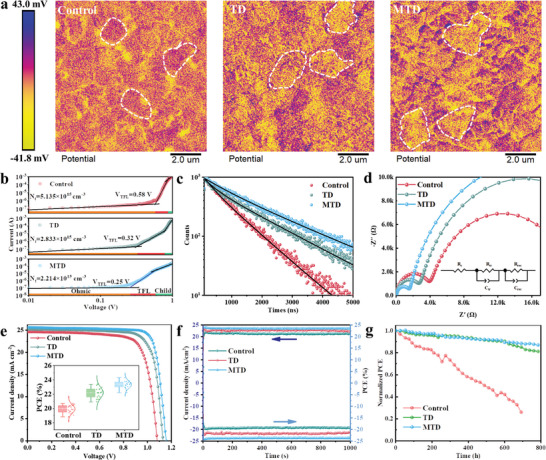
Photovoltaic performance and stability of PSCs. KPFM images of Control, TD, MTD perovskite films. b) Dark *I*–*V* curve of hole‐only devices (FTO/PTAA/Perovskite/Spiro‐OMeTAD/Au) assembled with Control, TD, MTD perovskite film, respectively. c) TRPL carrier decay curves of Control, TD, MTD perovskite films. d) Nyquist plots of Control, TD, MTD PSCs measured in the dark. The inset shows the equivalent circuit model. e) Reversed scanned *J*–*V* curves of champion PSCs. The inset shows the statistical distributions of PCE from 25 independent cells. f) Stabilized power output at the MPP voltage for 1000 s. g) Humidity stability test of the unencapsulated PSCs at 50 ± 10% RH and 25 ± 5 °C in the dark.

Compared with 3D perovskites, 2D perovskites possess significant advantages, such as suppressed ion migration, efficient carrier transport paths, and enhanced resistance to moisture and heat.^[^ ^51^, ^52^ ^]^ Herein, 2D phases passivated GBs rather than being incorporated into the perovskite lattice due to larger size of OA^+^, as validated by Kelvin probe force microscopy (KPFM) characterization, **Figure** **5**a. KPFM analysis indicated that the contact potential difference (CPD) of the GBs was lower than that of the grain interior for the Control film. By contrast, the GBs of TD and MTD films had higher CPD than the grain interior, ascribing to the low surface trap density and high carrier concentration after 2D phase treatment.^[^ ^53^ ^]^ Moreover, 2D phases had slight influence on the bandgap due to their distribution at GBs,^[^ ^29^, ^54^ ^]^ Figure S25a (Supporting Information). The ultraviolet photo‐electron spectroscopy (UPS) tests were employed to further illustrate the effect of 2D phases on the energy band (Figure S25b,c, Supporting Information). The inclusion of 2D perovskites increased the valence‐band maximum (VBM) and conduction‐band minimum (CBM) of perovskite from −5.76 and −4.23 eV to −5.69 and −4.16 eV, respectively. This result indicates better energy level alignment between the perovskite layer and HTL, which is beneficial for hole extraction.^[^ ^55^ ^]^ The space‐charge‐limited‐current (SCLC) technique was employed to measure the trap density (*N*_trap_) of perovskite films based on a hole‐only structure of ITO/PTAA/perovskites/ Spiro‐OMeTAD/Au. As seen in Figure 5b, the value of *N*_trap_ was 5.135 × 10^15^ cm^−3^, 2.833 × 10^15^ cm^−3^, 2.214 × 10^15^ cm^−3^ for the Control device, TD device, and MTD device, respectively. The decreased trap density was consistent with remarkable defect passivation and improved film quality, both of which were beneficial for enhancing device performance.^[^ ^28^, ^56^ ^]^ Time‐resolved photoluminescence spectroscopy (TRPL) was performed to investigate the influence of the 2D perovskite and MAI additive on charge recombination in the 3D *α*‐phase perovskite (Figure 5c). The TRPL spectra were fitted using the double exponential decay function: $y\;=A_{1}\;\exp\left(\frac{-t}{\tau_{1}}\right)+A_{2}\exp\left(\frac{-t}{\tau_{2}}\right)+y_{0}$,where *A*_1_ and *A*_2_ represent the relative amplitudes, τ_1_and τ_2_denotes carrier lifetimes of fast non‐radiative recombination and slow radiative recombination, respectively. The average carrier lifetime (τ_*avg*_) can be estimated by equation: $\tau_{avg}=\frac{\sum A_{i}{\tau_{i}}^{2}}{\sum A_{i}\tau_{i}}\;$.^[^ ^57^ ^]^ As listed in Table S1 (Supporting Information), the increased τ_*avg*_ of TMD film indicated that the traps were significantly reduced, which was attributed to the fact that the improved GBs and vertical crystal orientation resulted in a reduction in trap‐mediated bulk or surface recombination.^[^ ^58^ ^]^ The capacitance–voltage (*C*–*V*) curve was acquired to investigate the built‐in potential (*V*_bi_) of the perovskite film according to the Mott–Schottky relationship (Figure S26, Supporting Information).^[^ ^59^ ^]^ The *V*_bi_ values followed the order of MTD (0.94 V)>TD (0.89 V)>Control (0.83 V). Hence, more efficient charge separation and higher open‐circuit voltage (*V*_oc_) are expected for MTD device.^[^ ^60^ ^]^ Figure 5d presents Nyquist diagrams of the devices comprising Control, TD, and MTD films. The inset shows the equivalent circuit model, and the fitted impedance parameters are summarized in Table S2 (Supporting Information). The MTD device delivered the lowest series resistance (*R*_s_) and transfer resistance (*R*_tr_) as well as the highest recombination impedance (*R*_rec_), indicating that the charge carrier's recombination and defects formation occurred with greater difficulty in the defect‐heal MTD film.^[^ ^61^ ^]^ Solar cells with an n‐i‐p‐type structure of FTO/SnO_2_/Perovskite/Spiro‐OMeTAD/Au were constructed. Figure 5e presents the *J*–*V* curves of Control device, TD device, and MTD device under one sun illumination (AM 1.5G, 100 mW cm^−2^). The photovoltaic parameters are listed in Table S3 (Supporting Information). The Control cell exhibited a *PCE* of 20.24%, *V*_oc_ of 1.08 V, short‐circuit current density (*J*_sc_) of 24.64 mA cm^−2^, and fill factor (*FF*) of 76.07%. Among them, the best‐performing MTD device yielded the highest PCE of 24.19% with an enhanced *V*_oc_ of 1.16 V, a *J*_sc_ of 25.68 mA cm^−2^, and an *FF* of 81.20%, which was mainly caused by the enhanced crystallinity and more preferred orientation at the (100) facet. Furthermore, the statistical distribution of the PCE from 25 independent devices exhibited the same results (the inset of Figure 5e), definitely confirming the overwhelming superiority of the 2D‐induced 3D controlled growth strategy. By comparing the photovoltaic performance based on forward and reverse scan directions (Figure 5e; Figure S27, Supporting Information), MTD devices exhibited virtually negligible hysteresis, Table S3 (Supporting Information). By integrating the external quantum efficiency (EQE) over the AM 1.5G standard spectrum, the projected *J*_sc_ of Control device, TD device, MTD device was 23.87, 24.96, and 25.13 mA cm^−2^, respectively, which were quite close to those obtained from the *J*–*V* curves, Figure S28 (Supporting Information). The steady‐state PCE output tracking of the PSCs in Figure 5f yielded a PCE of 21.5% and 23.7% for the TD and MTD based PSCs, respectively, larger than that of the Control cell (19.3%), which further confirmed the reliability of the *J*–*V* characteristics. To assess the stability of PSCs, we measured first their humidity stability by storing the unencapsulated devices in the dark at 25 °C and 50 ± 10% relative humidity, Figure 5g. The PCE of the MTD cell decreased by only 13% after 800 h aging, whereas a serious PCE decrease was observed in the Control cell, with only 25% of the initial value left after ≈700 h. We attributed the greater stability of MTD cell to the water‐resistance role of OA^+^ in 2D phases that existed at GBs,^[^ ^62^ ^]^ as confirmed by the higher contact angles for TD (68.6°) and MTD (68.9°) films, than 40.5° for the Control film (Figure S29, Supporting Information). We further investigated the long‐term operational stability of PSCs by aging the unencapsulated devices under a nitrogen atmosphere, using maximum power point (MPP) tracking under a continuous light irradiation with a white LED lamp (100 mW cm^−2^). As observed from Figure S30 (Supporting Information), the PCE of the Control cell decreased by ≈35.9%, while the TD cell lost ≈19.3% of its initial efficiency. The best stability of preserving 86.1% of initial PCE is observed for the MTD cell, which partially originates from the vertically oriented crystal growth, improved crystal quality and film uniformity.

## 3 Conclusion

In this work, we gained insight into the roles of MAI additive in RP‐type 2D template‐assisted controlled crystallization of *α*‐FAPbI_3_ perovskite films by in situ optical diagnostics during spin coating and subsequent thermal annealing. The 2D template of n = 2 (OA_2_MAPb_2_I_7_), arising from the transition of n = 1 2D (OA_2_PbI_4_), preferentially form in the initial stage of crystallization due to a low formation energy barrier. However, 2D templates were inevitably destroyed when volatile MACl left the bulk film during thermal annealing, resulting in void and volume collapse. We reveal that small amounts of MAI additive make the 2D template structure more stable, leading to more controlled crystallization and a strong degree of preferential orientation during the dissolution‐recrystallization process. The high‐quality mixed‐dimensional perovskite film delivered a high PCE of 24.19% together with improved humidity and operational stability. We believe that our new finding will help researchers to better understand the role of 2D phases in the formation mechanism and crystallization pathways of *α*‐FAPbI_3_ perovskite film. It is anticipated that the 2D/3D heterostructure will be one of the important strategies to achieve high‐performance PSCs.

## Conflict of Interest

The authors declare no conflict of interest.

## Supporting information

Supporting Information

## Data Availability

The data that support the findings of this study are available from the corresponding author upon reasonable request.
