# Supplementary material for: New Insights into MAI Additives in 2D‐Assisted 3D Controlled Crystallization Toward High‐Quality α‐Phase FAPbI3 Perovskites
Source: Adv Sci (Weinh). 2024 Aug 6;11(38):2402065. doi: 10.1002/advs.202402065 (PMC11481258; doi:10.1002/advs.202402065)
Supplement: Supplementary file 1 — Supporting Information [file ADVS-11-2402065-s001.pdf]

ADVANCED SCIENCE
Open Access

Supporting Information

for *Adv. Sci.*, DOI 10.1002/advs.202402065

New Insights into MAI Additives in 2D-Assisted 3D Controlled Crystallization Toward High-Quality α-Phase $FAPbI_3$ Perovskites

*Tao Liu, Meichen Hou, Wending Hao, Shitong Du, Wenbin Yang, Yihui Yuan and Ning Wang**

Supporting Information

New Insights into MAI Additives in 2D-Assisted 3D Controlled Crystallization toward High-Quality α-phase $FAPbI_3$ Perovskites

*Tao Liu,[#] Meichen Hou,[#] Wending Hao, Shitong Du, Wenbin Yang, Yihui Yuan, Ning Wang**

* Corresponding author

[#] These authors contributed equally to this work.

Prof. T. Liu, M. Hou, W. Hao, S. Du, W. Yang, Prof. Y. Yuan, Prof. N. Wang

State Key Laboratory of Marine Resource Utilization in South China Sea

Hainan University,

Haikou 570228, P. R. China

E-mail: wangn02@foxmail.com

Supporting Information

Table of Contents

1. **Materials** 1

2. **Experiment Section** 1

3. **Characterization Section** 3

4. **Figures** 7

5. **Tables** 23

6. **References** 24

1. Materials

N,N-Dimethylformamide (DMF; 99.8%), Dimethyl sulfoxide (DMSO; >99.9%), chlorobenzene (CB; 98.0%), Acetonitrile (ACN; 99.8%) were purchased from Sigma-Aldrich. Tin oxide (SnO_2; 15% in H_2O) was purchased from Alfa Aesar. Methylammonium chloride (MACl; ≥ 99.99%), Formamidinium iodide (FAI, ≥ 99.99%) and Methylammonium iodide (MAI; ≥ 99.99%) were purchased from GreatCell Solar. Lead iodide (PbI_2; 99.999%) and octylamine hydroioiodide (OAI) were purchased from Sigma-Aldrich. 2,2',7,7'-tetrakis (N,N-di-pmethoxyphenylamine)−9,9'-spirobifluorene (Spiro-OMeTAD; >99.8%) were purchased from Borun New Materials Technology. Phenylethylamine hydroiodide (PEAI; 99.5%), lithiumbis (trifluoromethanesulfonyl) imide salt (Li-TFSI; ≥ 99%), FK209-Co (III)-TFSI (≥ 98%) and 4-tert-butyl-pyridine (TBP; > 96.0%) were purchased from Xi'an Yuri Solar Corp. Isopropyl alcohol (IPA; 99.7%) was purchased from Xi long Scientific. All reagents were used as received without any purifications.

2. Experiment Section

Synthesis of 2D $OA_2MA_{n-1}Pb_nI_{3n+1}$ single crystal

The 2D $OA_2MA_{n-1}Pb_nI_{3n+1}$ perovskite (n=1, 2, 4) were synthesized by the solution cooling precipitation method reported previously. PbI_2, MAI and OAI were mixed in appropriate stoichiometry in HI solution (57 wt% in H_2O) and H_3PO_2 solution (50 wt% in H_2O). The mixed solution was stirred magnetically at 180℃ and allowed to dissolve completely. Then, the temperature was lowered to room temperature to obtain millimeter-scale crystals. The final lamellar single crystals were obtained by rising with CB several times and then drying in an

oven at 50 ºC for 12 h.

Synthesis of $FAPbI_3$ powder

Formamidinium lead triiodide ($FAPbI_3$) black powder was synthesized by adding FAI (1.12 g) and of PbI_2 (3 g) (1:1 molar ratio) in ACN (5 ml). The mixed solution was heated to 150 °C and then stirred for 120 min. Subsequently, the precipitate was collected by filtration. The final product was dried in air on a hot plate at 150 °C for 30 min and then left overnight in a vacuum chamber at 80 °C.

Device fabrication

Fluorine-doped tin oxide (FTO) coated conductive glass was sequentially cleaned with detergent, deionized (DI) water, acetone, and IPA for 15 min, respectively. Then, UV ozone treatment was performed for 15 min prior to use. The diluted SnO_2 colloid solution (SnO_2:DI water = 1:3, volume ratio) was spin-coated on the as-cleaned FTO glass substrate at 4000 rpm for 30 s, and then subjected to a 150 °C annealing for 30 min on a hotplate. The precursor solution of the control perovskite was prepared by dissolving 1.4 M pre-synthesized $FAPbI_3$, PbI_2 (15 mol%) and MACl (35 mol%) in a mixed solvent of DMF:DMSO=8:1 (molar ratio). As for the TD perovskite, 2D single-crystals (0.2 mol%) were added into control perovskite precursor solution. For the target MTD perovskite, 2D single-crystals (0.2 mol%) and MAI (0.2 mol%) were added into control perovskite precursor solution. The precursor solution of three perovskites (Control, TD, MTD) was filtered with a 0.22 μm PTFE filter prior to use. For each perovskite sample, 60 μL of the filtered precursor solution was spin-coated onto FTO/SnO_2 substrate at 5000 rpm for 40 s. During spin coating step, 200 μL of CB was quickly dripped on the rotating substrate (5000 rpm) after spinning for 10 s. Then the film was dried

on a hotplate at 150 °C for 15 min to form the required α-phase $FAPbI_3$. After cooling to room temperature, the perovskite film was spin coated with 5 mg/mL of PEAI dissolved in IPA at 4,000 rpm. Thereafter, the Spiro-OMeTAD layer was subsequently deposited on the as-prepared perovskite layer by spin-coating of 60 μL of stock solution, which was composed of 90 mg Spiro-OMeTAD in 20 μL Li-TFSI solution (520 mg Li-TFSI in 1 mL ACN), 8 μL FK209- Co(III)-TFSI solution (300 mg in 1 mL ACN), 36 μL TBP and 1 mL CB, at 4000 rpm for 30 s. Finally, Au electrode with a thickness of ca. 80 nm was evaporated on the top of the Spiro-OMeTAD layer using a thermal evaporator.

3. Characterization Section

Material characterization

X-ray diffraction (XRD) data were collected from a Rigaku D-Max 2200 with a radiation source of Cu $K\alpha_1$ line (λ = 0.1542 nm). Grazing-incidence wide-angle x-ray scattering (GIWAXS) measurements were performed on a Xeuss 3.0 at an incident angel of 0.2°. The optical absorption spectra were acquired by an ultraviolet/visible/near-infrared (UV−vis−NIR) spectrophotometer (PerkinElmer LAMBDA 750). Ultraviolet photoelectron spectroscopy (UPS) characterizations are conducted in a ThermoFisher Nexsa surface analysis system equipped with a He discharge lamp ($h\upsilon$= 21.22 eV) and a monochromatic Al-Kα X-ray gun ($h\upsilon$= 1486.68 eV). The surface and cross-section morphologies of perovskite films were acquired by field-emission scanning electron microscope (SEM) (thermoscientific Verios G4 UC). The morphological characteristics of the perovskite films during crystallization were detected and revealed by metalloscope (LW200-3JT) in real time. The surface potential of the perovskite film was acquired by the scanning Kelvin probe force microscopy (SKPM) (Bruker

Dimension Icon). The size distribution was measured by laser light laser particle size analyzer (Zetasizer Nano ZSE). The water contact angles on the surface of perovskite films were measured by using a contact angle meter. Steady-state photoluminescence (PL) and time-resolved photoluminescence (TRPL) were measured using a fluorescence spectrometer (Edinburgh FLS1000). TRPL decay transient's data were collected at 809 nm using excitation with a 468 nm light pulse. In-situ PL data were acquired by a home-built equipment, including an excitation system, an optical fiber system and a detector system. The excitation laser (405 nm, max = 20 W) hit the perovskite surface through an optical fiber. The emitted light from the sample was received by a spectrophotometer (Ocean Optics USB2000) through another optical fiber. A 500 nm long pass filter is applied in the light pass to the spectrophotometer. In situ PL measurements were conducted in a dark environment at 25 °C, 15-20% humidity. Electrochemical Impedance Spectroscopy (EIS) and Mott−Schottky data of devices were collected on Chen Hua CHI760E electrochemical workstation. The trap-state density (N_t) was estimated using hole-only devices with the configuration of FTO/PTAA/Perovskite/Spiro-OMeTAD/Au. The devices were measured from -2 V to 2 V in the dark. The observed response was analyzed according to space-charge-limited current (SCLC) theory. [1] N_{trap} was calculated according to the equation:

$$N_{trap} = \frac{2V_{TFL}\varepsilon_0\varepsilon_r}{eL^2} \quad (1)$$

where V_{TFL} is the trap-filled-limit voltage, ε_0 is the vacuum permittivity (ε_0 =8.854×10^{-14} F/cm), e is the electron charge (e=1.602×10^{-19} C) and L is the thickness of the perovskite film (L≈600 nm). The dielectric constant was calculated using the following equation:[2]

$$\varepsilon_r = \frac{Cd}{A\varepsilon_0} \quad (2)$$

where *C* is capacitance at high frequency (~10^4 Hz) and *A* is area.

Photovoltaic performance characterization

Current density-voltage (*J-V*) curves were recorded on a Keithley 2400 source meter under a simulated one sun (AM1.5 G, 100 mW cm^{-2}) illumination using a solar simulator (Newport Oriel Sol3A, Class AAA) equipped with 450 W Xenon lamp (Newport 6280NS). The light intensity was adjusted by NREL-calibrated silicon solar cell. The *J–V* curves of all devices were measured by masking the active area with an aperture area of 0.096 cm^2 at room temperature (ca. 25°C) in air. A voltage scan was performed from 1.2 to −0.2 V then back to 1.2 V with a step voltage of 20 mV. The incident photon-electron conversion efficiency (IPCE) was measured by a Quantum Efficiency System (Zolix solar scan 100) equipped with 150 W xenon lamp. The maximal steady-state photocurrent output for devices and their corresponding power output were measured by a constant voltage bias near the maximum power point (MPP). Moisture stability was recorded by storing the unencapsulated devices in ambient air (50±5% RH, room temperature). The operational stability tests were carried out at the MPP tracking for the unencapsulated cells under AM 1.5G white-LED solar simulator without the UV part in N_2 atmosphere.

Computational details

Density functional theory (DFT) calculations are performed using the Vienna ab initio simulation software (VASP). [3,4] To simulate electron exchange-related interactions, the generalized gradient approximation Perdew–Burke–Ernzerhof (PBE) [5] functional is utilized. The projection enhanced wave (PAW) approach [6,7] is used for electron–ion–nucleus interactions. The valence electrons were taken into account using a plane wave basis set with

a kinetic energy cutoff of 520 eV. First-principle calculations employed the ultra-soft pseudopotentials generated by the CASTEP on-the ultrasoft generator. And the Algorithm is BFGS. The electronic energy was considered self-consistent when the energy change was smaller than 10^{-6} eV. A geometry optimization was considered convergent when the energy change was smaller than 0.02 eV. The Brillouin zone integration is performed using 2×2×1 Monkhorst-Pack k-point sampling for a structure. The formation energies (E_f) of different species were computed using the chemical potentials for each constituent atom, determined by solving the set of linear equations describing the contributions from the starting species in the pristine derivative. Finally, we define the E_f per atom of a compound

$$E_f = E_{total} - N_{Pb}\mu_I - N_C\mu_C - N_H\mu_H \quad (3)$$

where E_{total} is the total DFT energy of a given structure, and μ{X,Pb,I,C,N,H} are the chemical potentials of the constituent atomic species, n{X,Pb,I,C,N,H} are the number of the constituent atomic species.

4. Figures

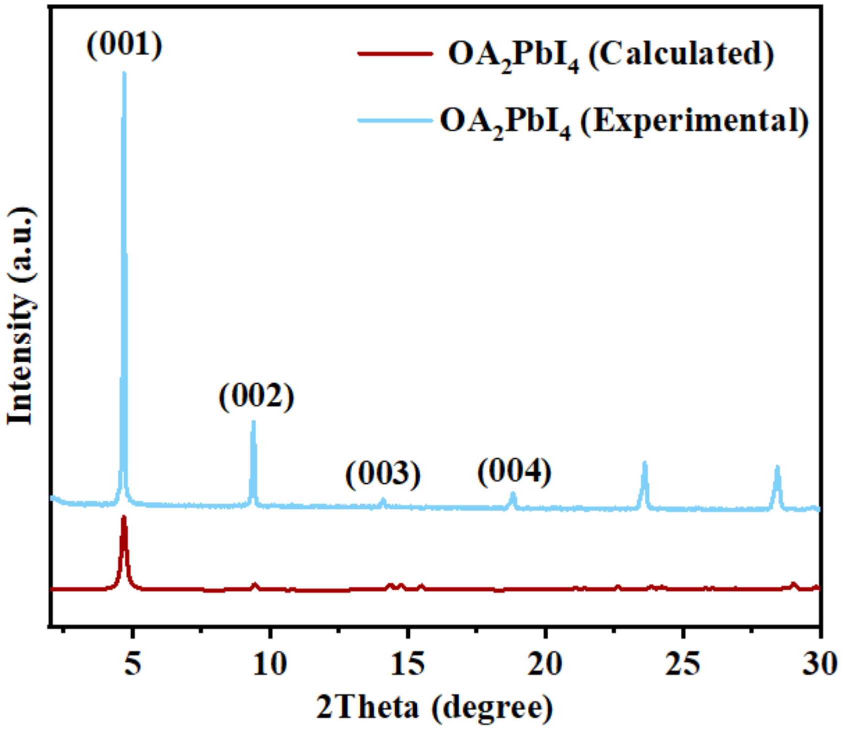

Figure S1. XRD patterns of 2D RP phase OA_2PbI_4 (n=1) single crystals.

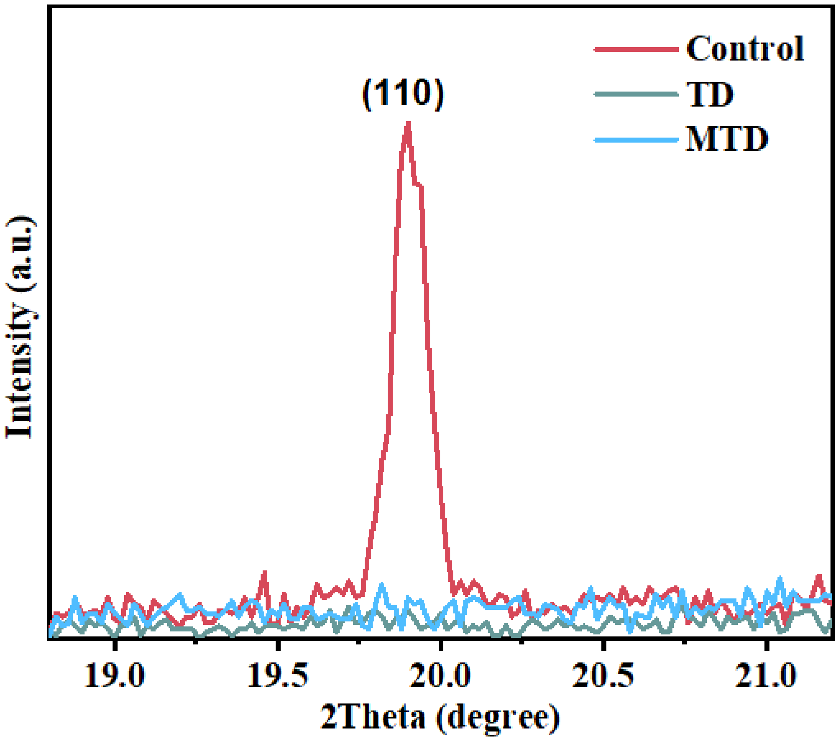

Figure S2. Local magnification XRD patterns in Figure 1b at 2θ≈20°.

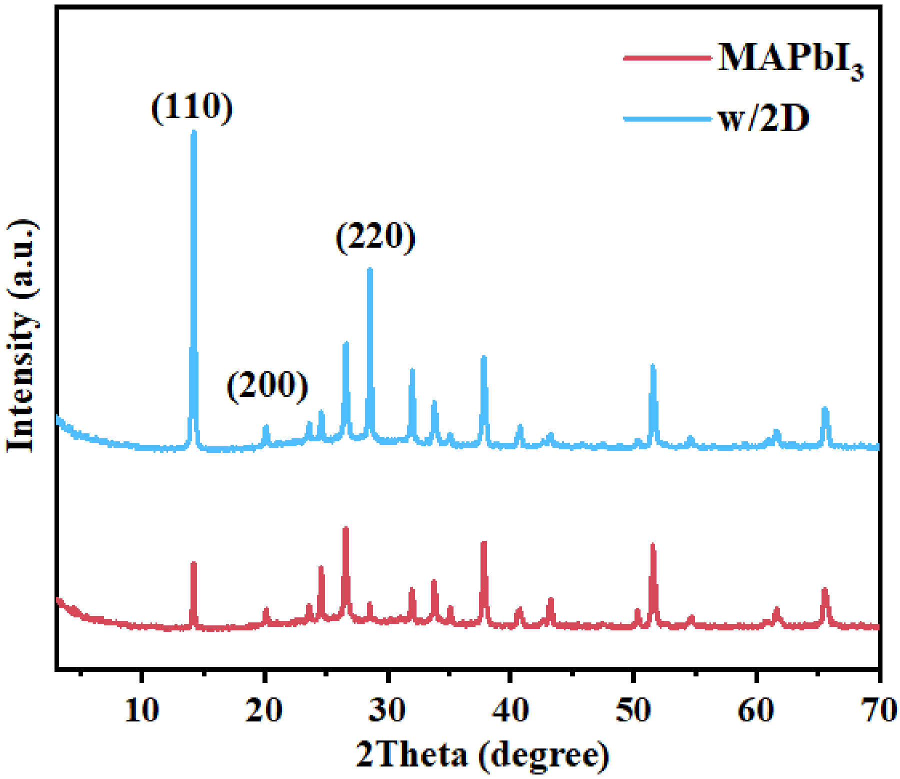

Figure S3. XRD patterns of pristine $MAPbI_3$ and 2D OA_2PbI_4 incorporated $MAPbI_3$ (w/2D).

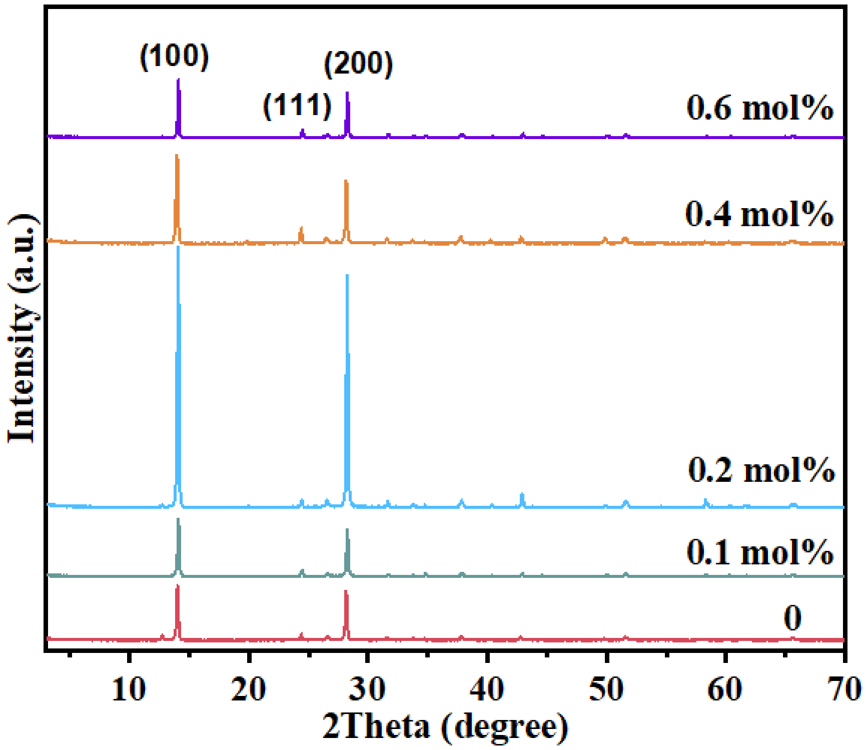

Figure S4. XRD patterns of MTD perovskite films with different concentrations of 2D OA_2PbI_4. The mole ratio of MAI to OA_2PbI_4 remains to be 1:1 for different concentrations.

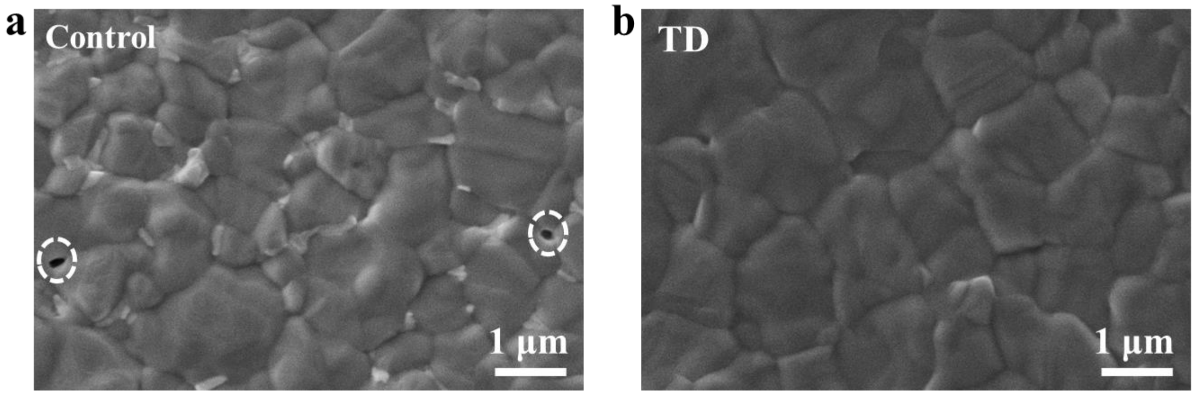

Figure S5. Top-view SEM images of a) Control and b) TD perovskite films.

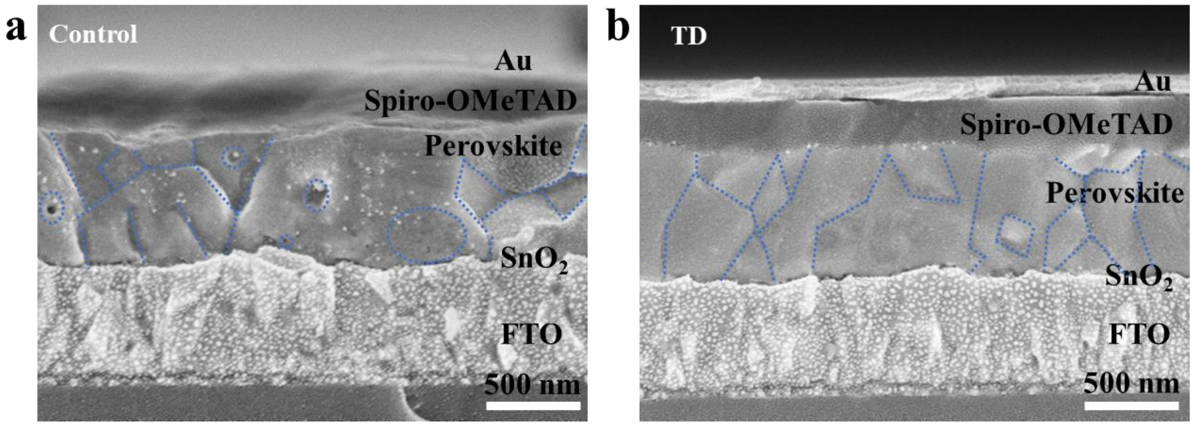

Figure S6. Cross-section SEM images of devices assembled with a) Control and b) TD perovskite films.

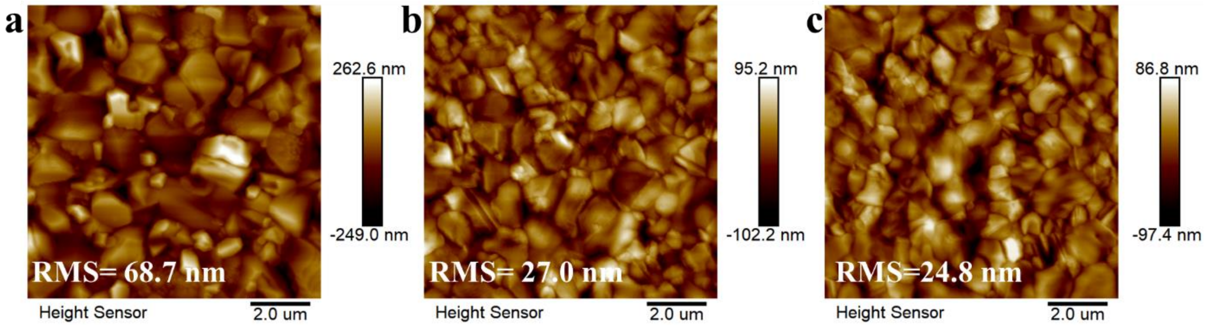

Figure S7. Atomic-force micrographs of a) Control, b) TD, c) MTD perovskite films.

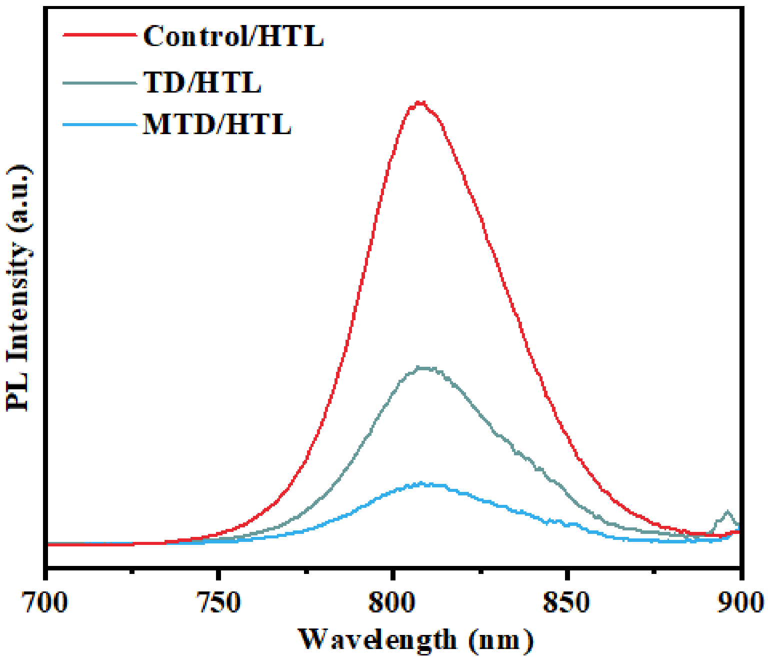

Figure S8. Steady-state PL spectra of Control/HTL, TD/HTL, MTD/HTL.

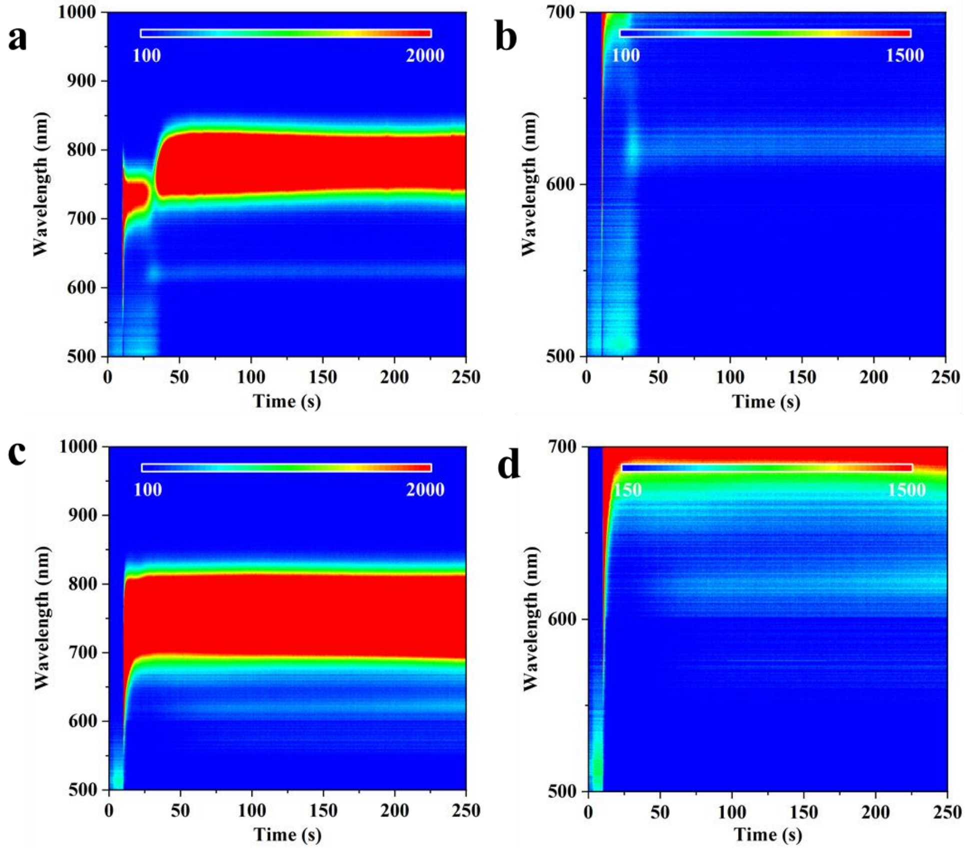

Figure S9. In situ PL mapping images of TD (a, b) and MTD (c, d) perovskite films during spin coating. For clear observation, the added concentration of 2D single crystals was 2 mol%.

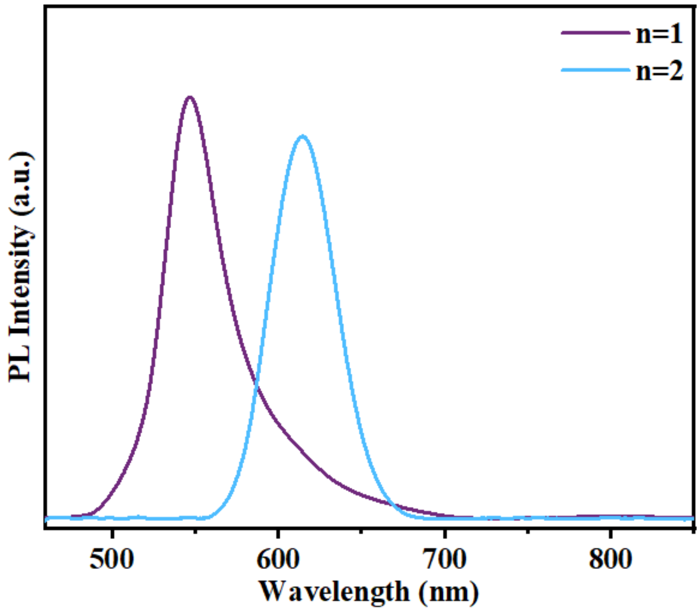

Figure S10. Steady-state PL spectra of n=1 2D (OA_2PbI_4) and n=2 2D ($OA_2MAPb_2I_7$) single crystals.

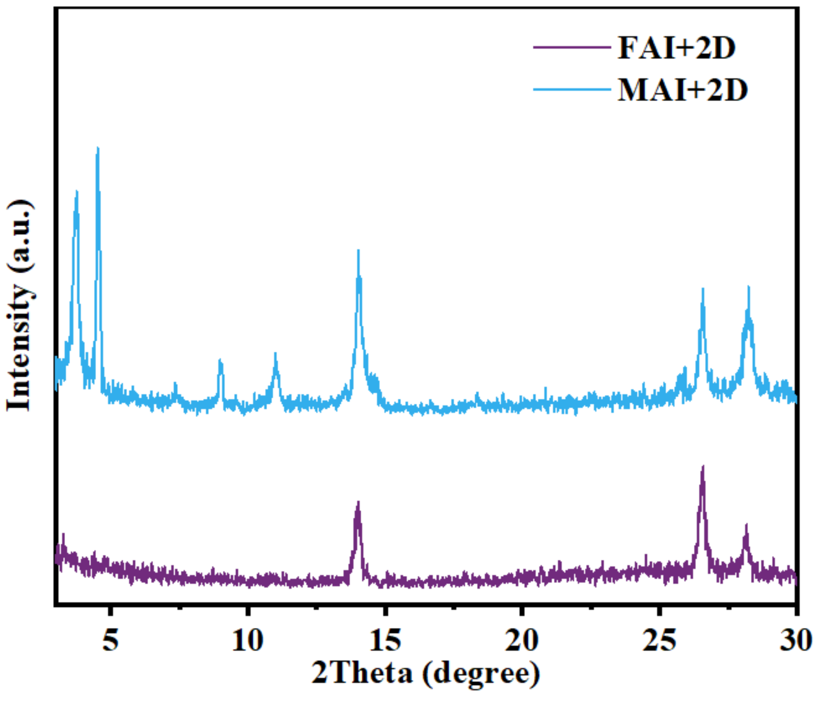

Figure S11. XRD patterns of FAI film with OA_2PbI_4 (FAI/2D) and MAI film with OA_2PbI_4 (MAI/2D) after annealing at 150 ºC.

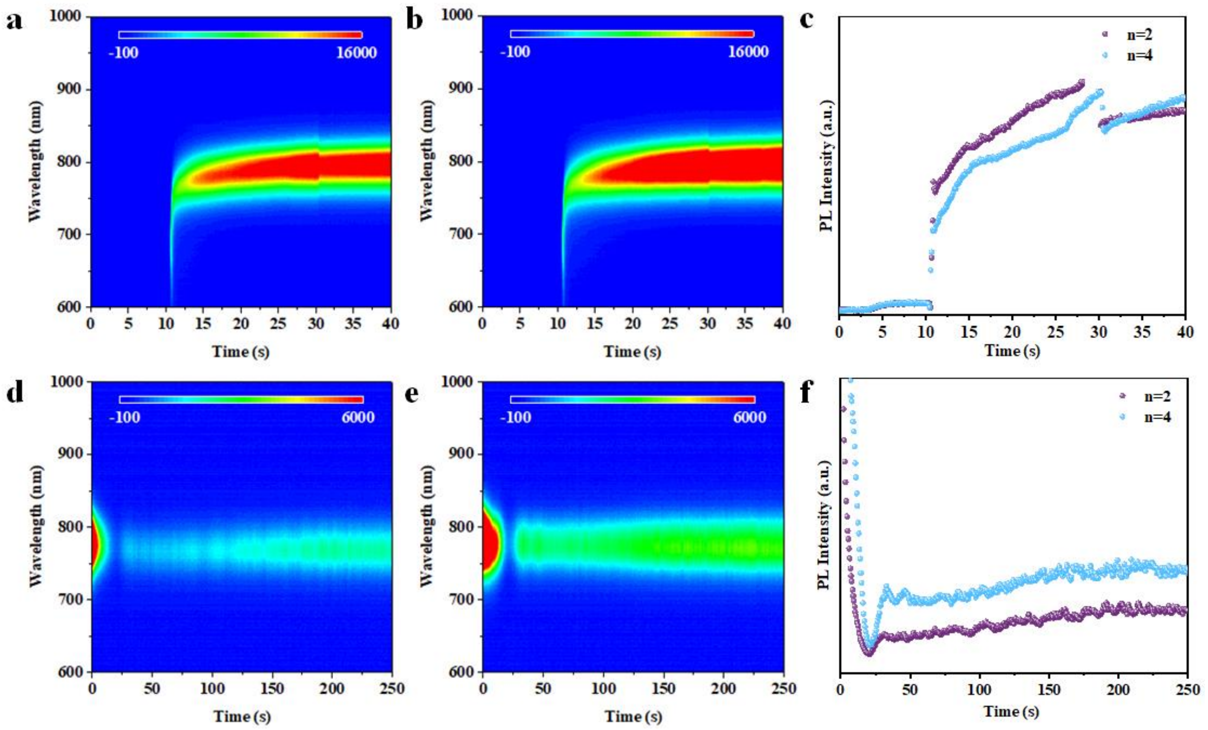

Figure S12. In situ PL mapping images of perovskite films with n=2 2D ($OA_2MAPb_2I_7$) (a, d) and n=4 2D ($OA_2MA_3Pb_4I_{13}$) (b, e) during spin coating (a, b) and annealing (d, e). The extracted PL peak intensity of perovskite films c during spin coating and f annealing.

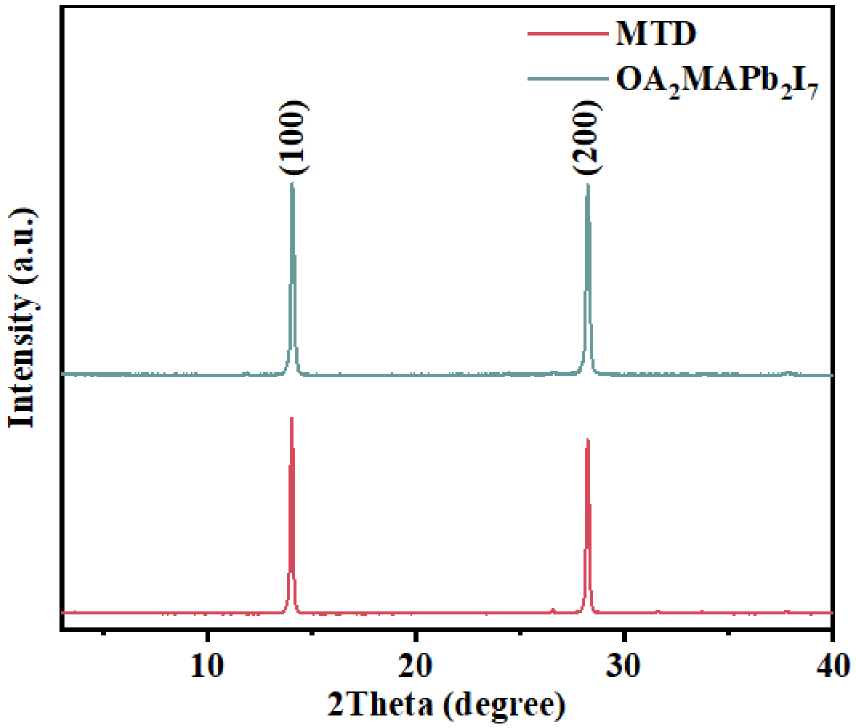

Figure S13. XRD patterns of MTD perovskite film and $OA_2MAPb_2I_7$ incorporated perovskite film.

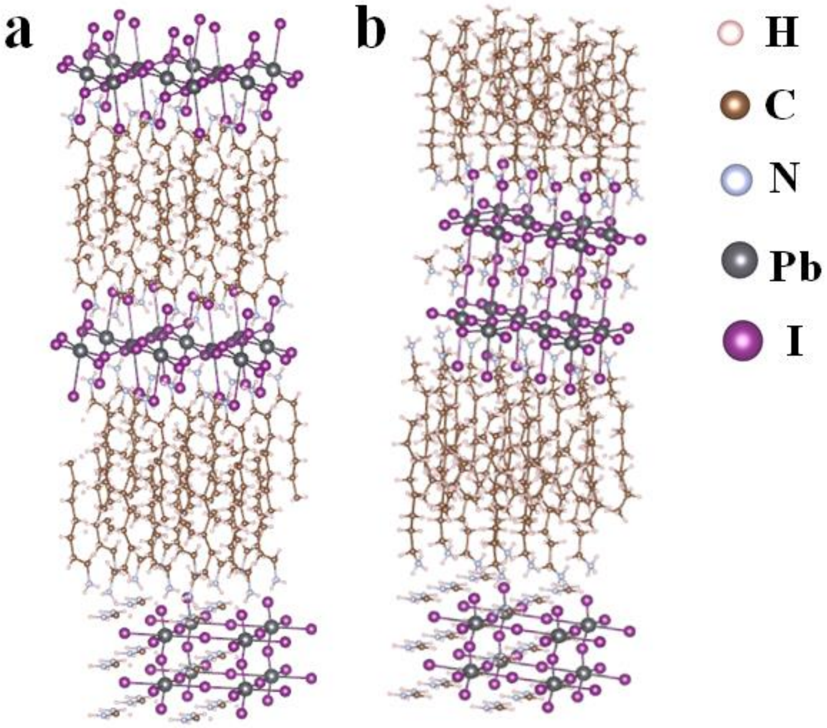

Figure S14. Molecular structure models of $FAPbI_3$ grown on a) n=1 2D ($FAPbI_3$/n=1) and b) n=2 2D ($FAPbI_3$/n=2).

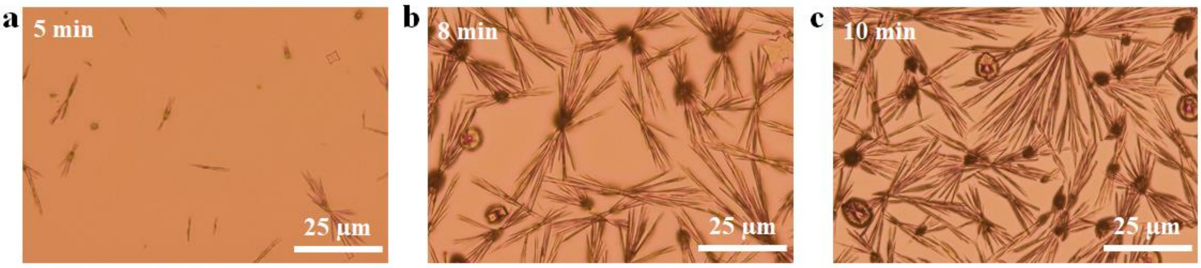

Figure S15. Optical microscope images of wet Control perovskite without dripping the antisolvent over time.

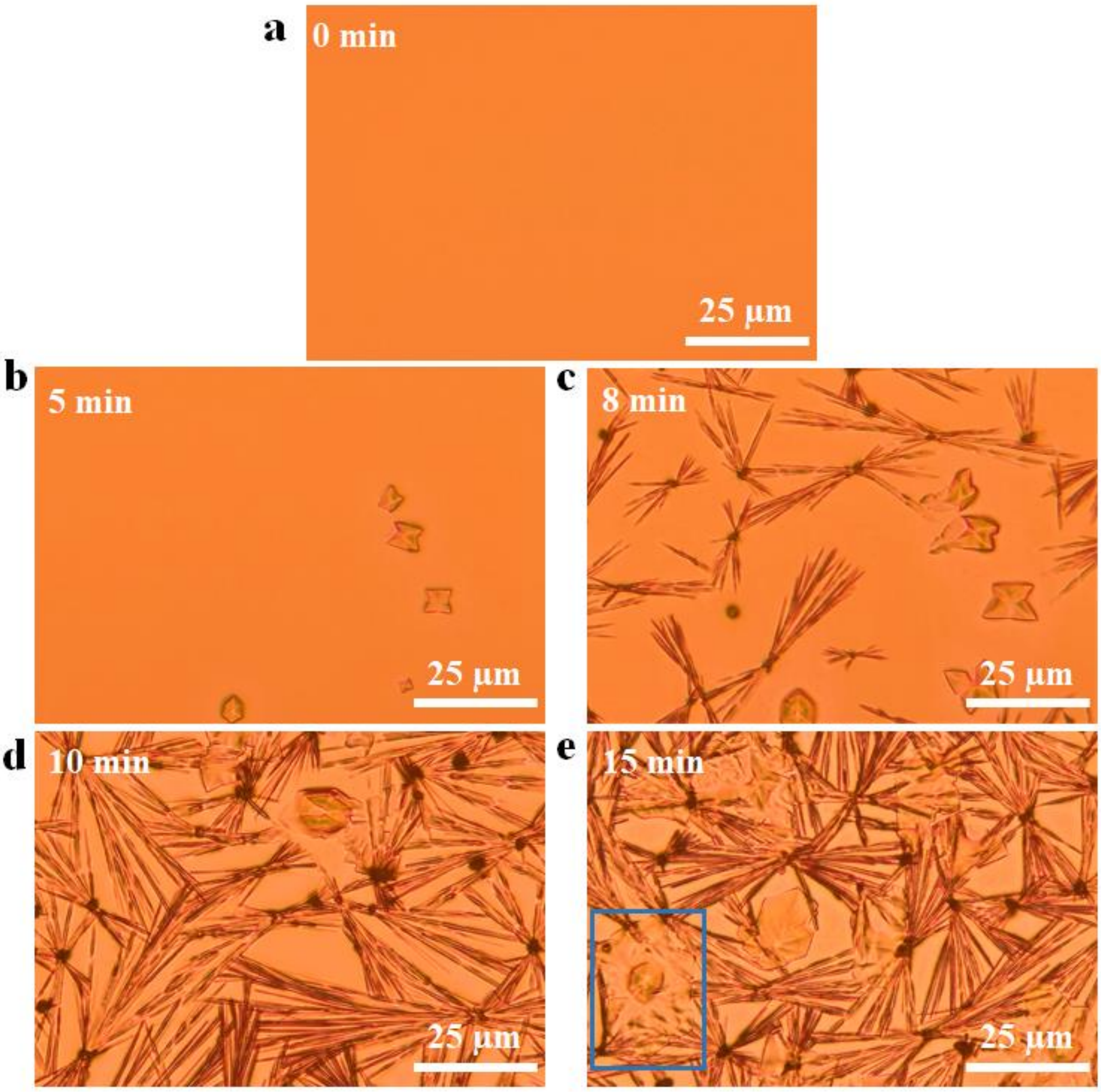

Figure S16. Optical microscope images of wet TD perovskite without dripping the antisolvent over time.

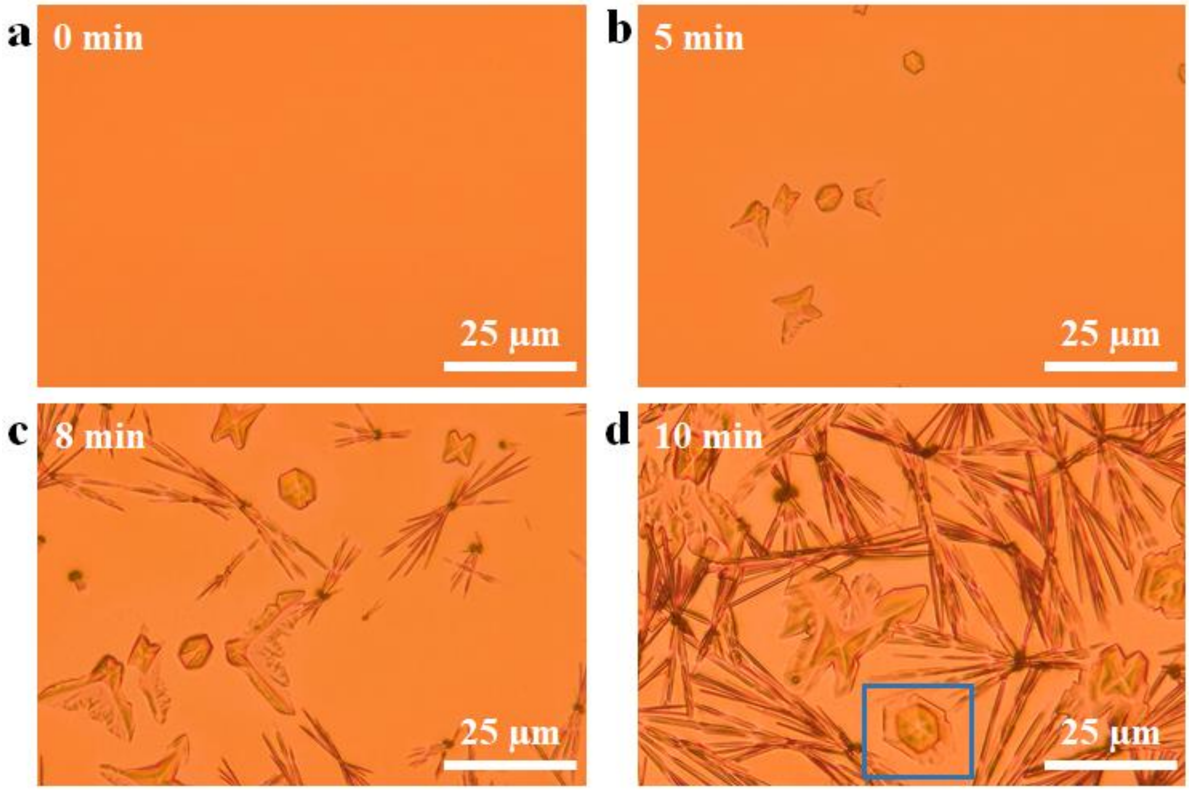

Figure S17. Optical microscope images of wet MTD perovskite without dripping the antisolvent over time.

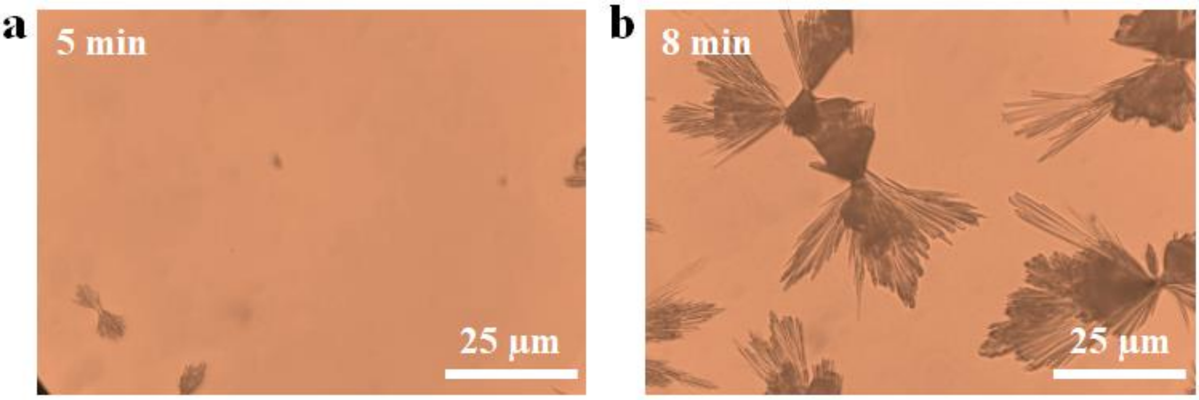

Figure S18. Optical microscope images of wet $MAPbI_3$ perovskite without dripping the antisolvent over time.

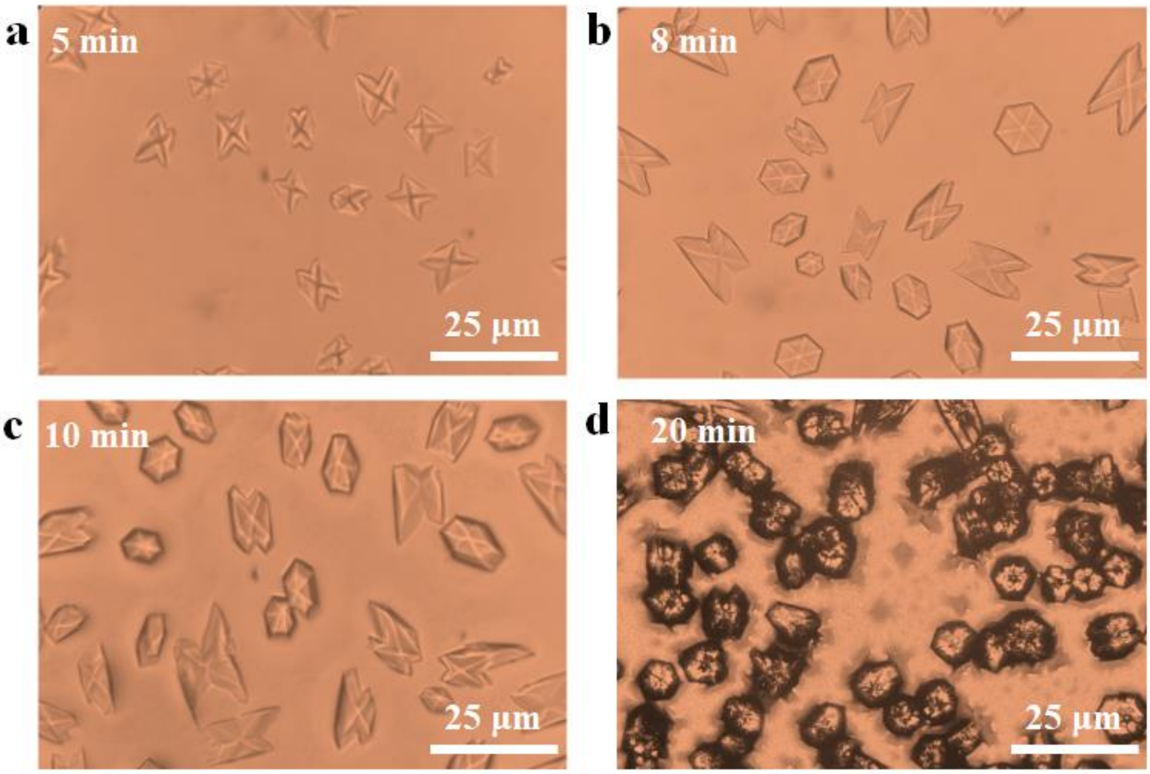

Figure S19. Optical microscope images of wet $FAPbI_3$ perovskite films without dripping the antisolvent over time.

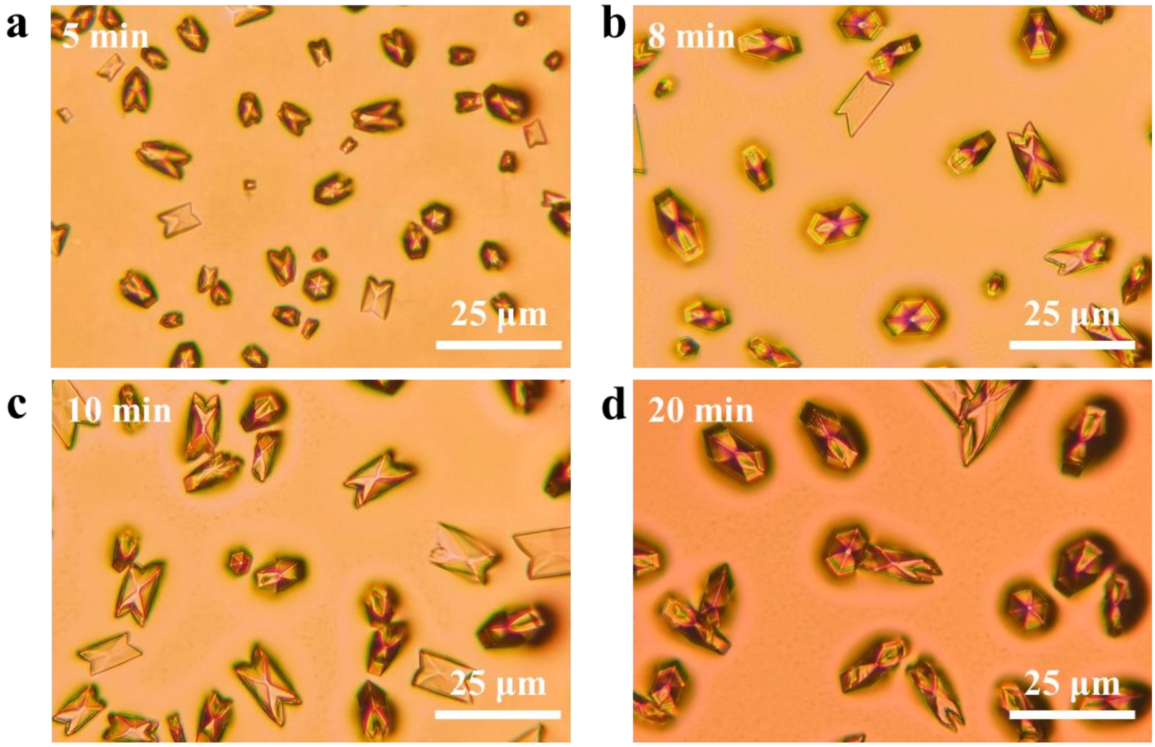

Figure S20. Optical microscope images of wet $FAPbI_3$ perovskite films containing n=1 2D without dripping the antisolvent over time.

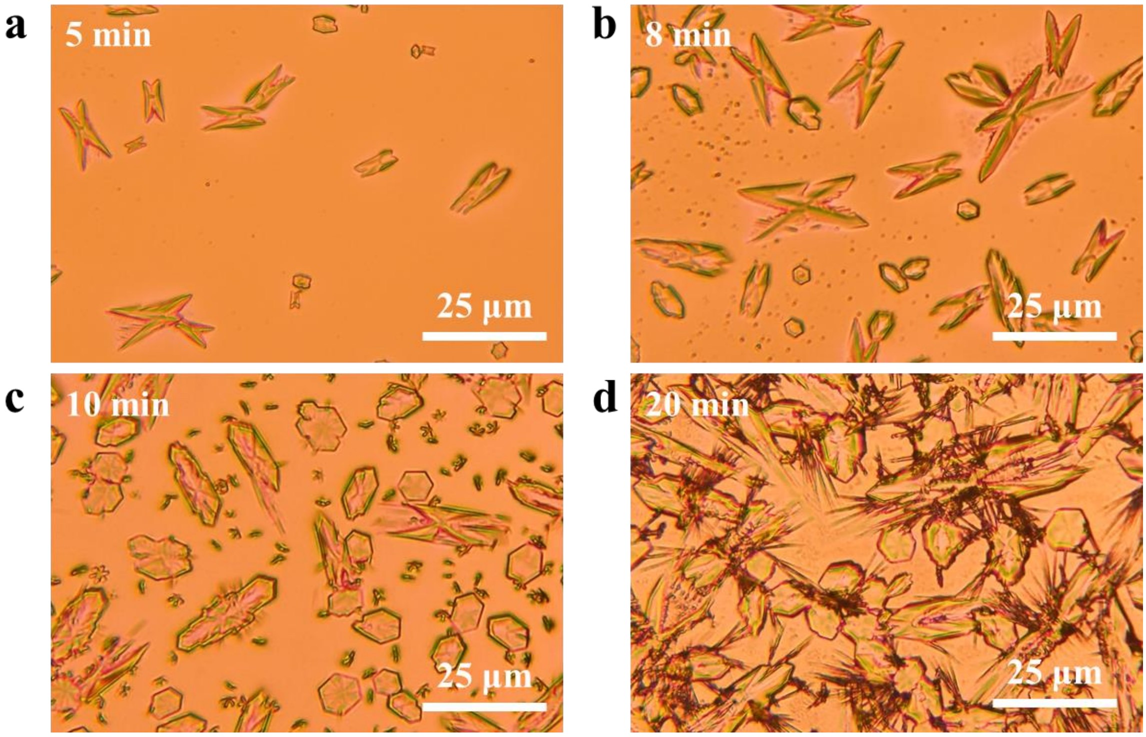

Figure S21. Optical microscope images of wet $FAPbI_3$ perovskite films containing MAI without dripping the antisolvent over time.

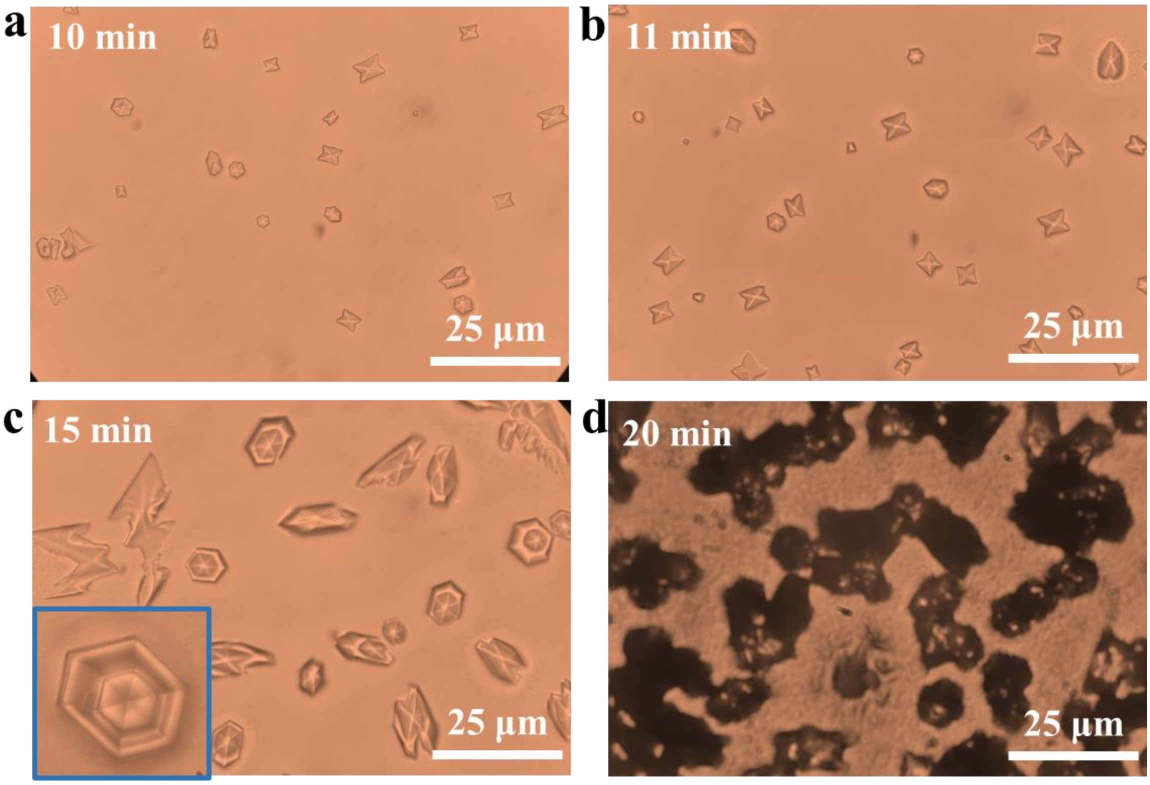

Figure S22. Optical microscope images of wet MTD perovskite (not containing MACl) without dripping the antisolvent over time. The inset in Figure S19c shows an enlarged image.

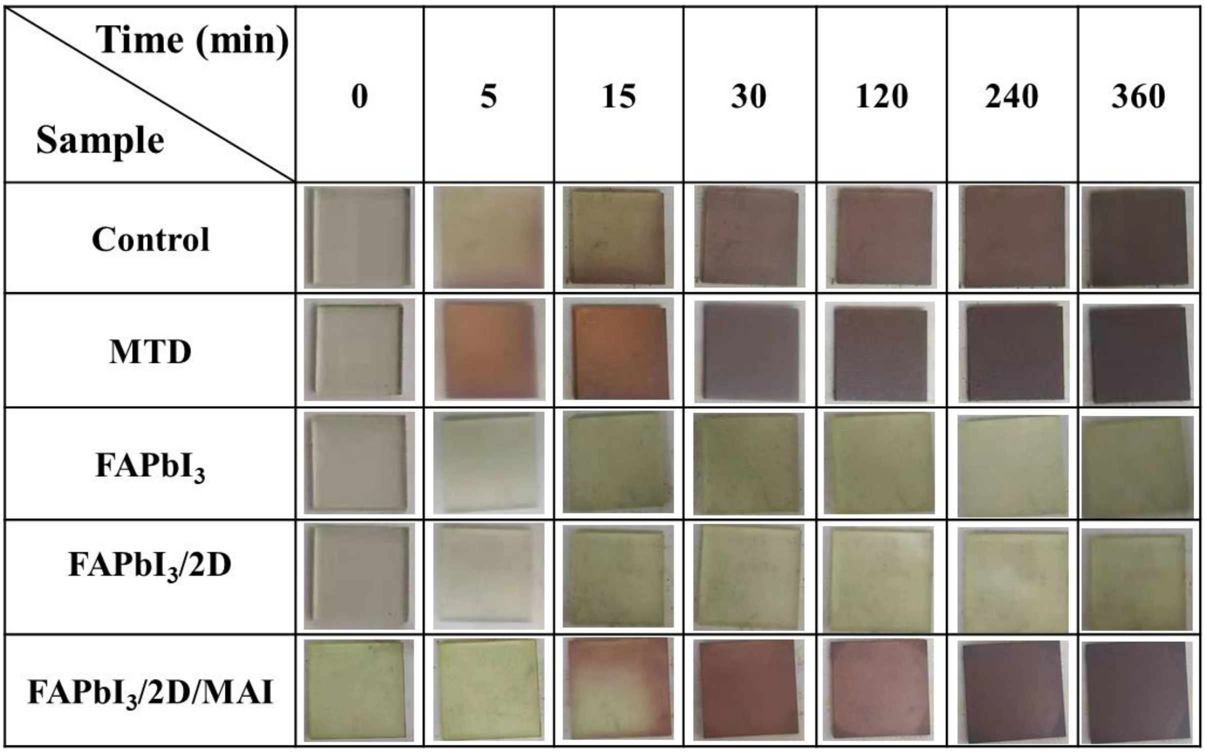

Figure S23. Digital photographs of the deposited perovskite films without dripping the antisolvent at room temperature.

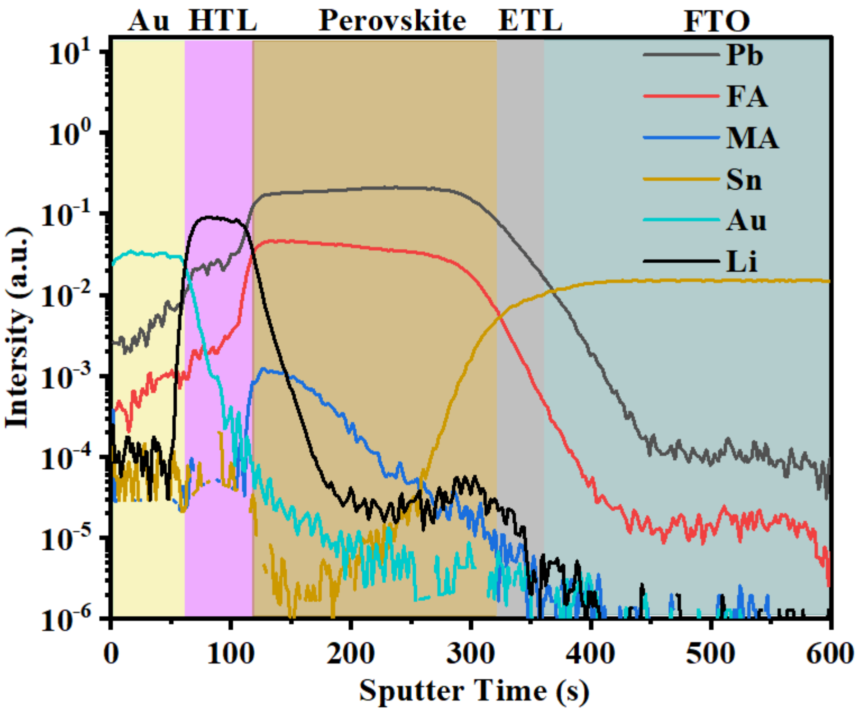

Figure S24. TOF-SIMs of MTD-based device.

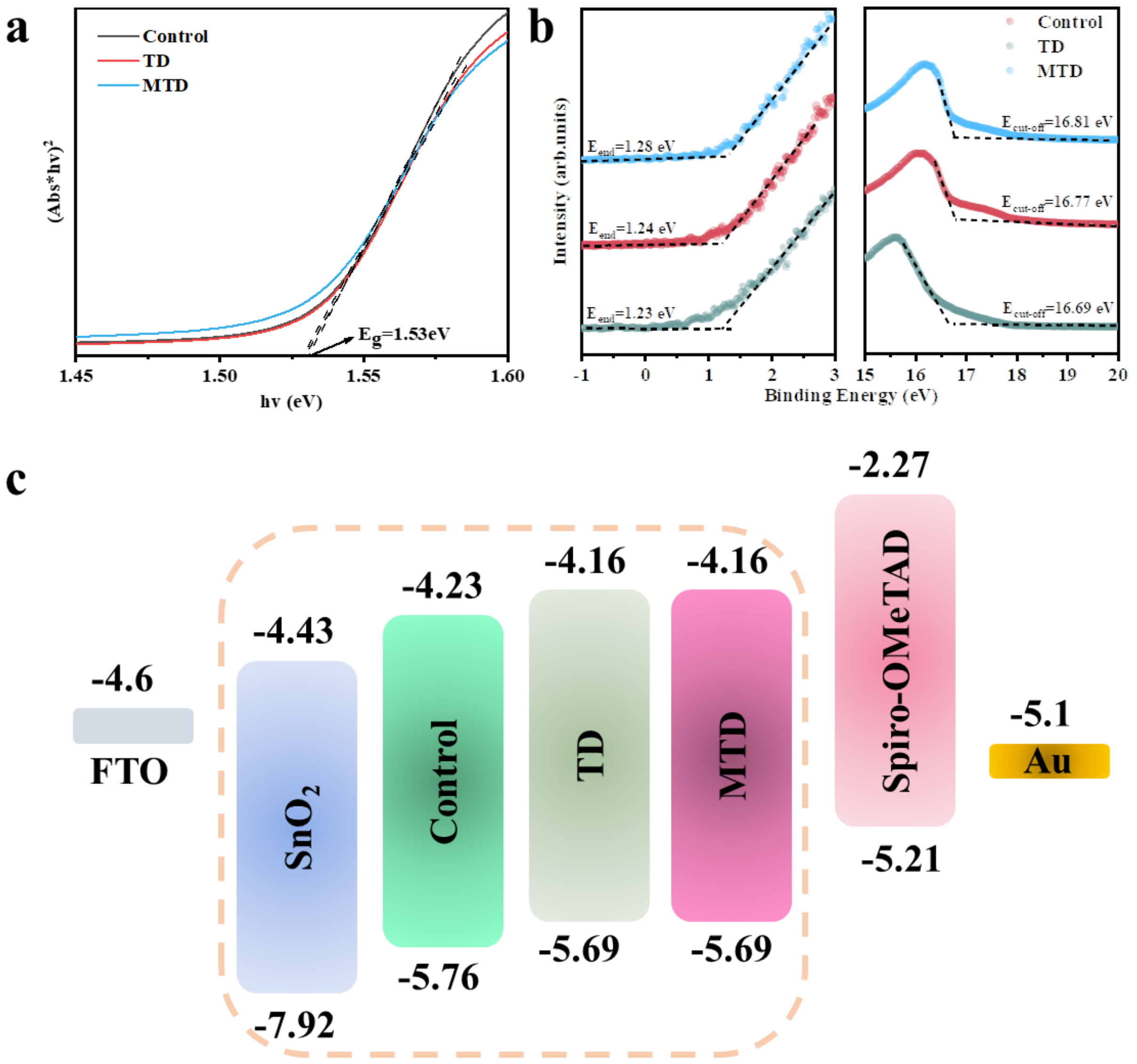

Figure S25. a) The relationship between $(\alpha hv)^2$ and photon energy (hv) for Control, TD and MTD perovskite films. b) The secondary electron cut-off region ($E_{cut\text{-}off}$) and the valence band region (E_{end}) of UPS spectra for Control, TD and MTD perovskite films. c) Energy level diagram of PSCs assembled with Control, TD and MTD perovskite films.

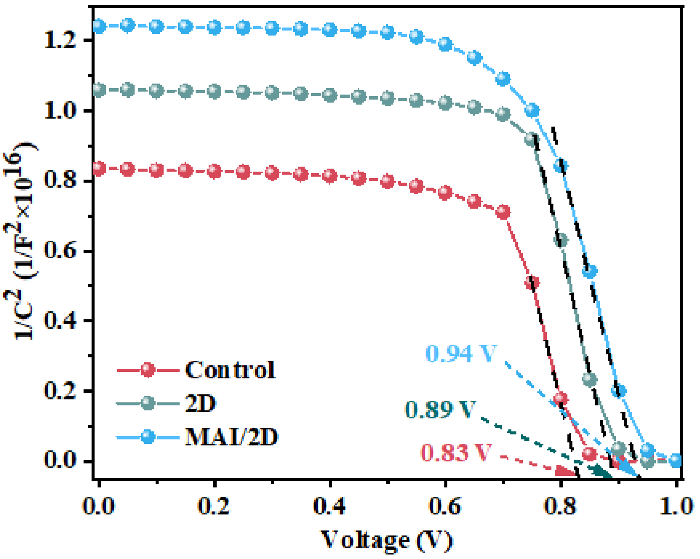

Figure S26. Mott−Schottky plots of PSCs assembled with Control, TD and MTD perovskite films.

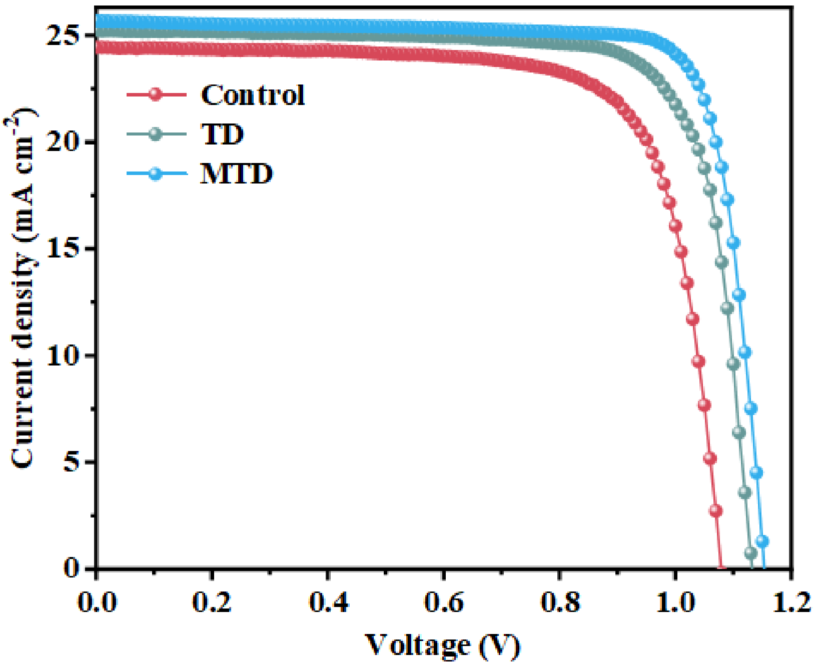

Figure S27. Forward scanned *J-V* curves of champion PSCs.

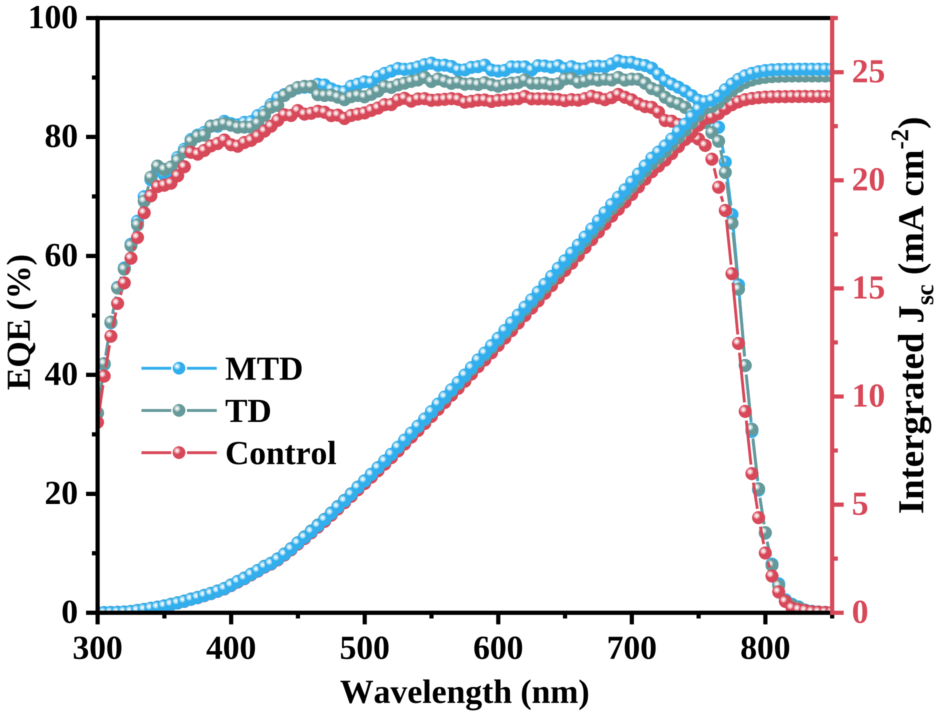

Figure S28. IPCE spectra of the best-performing devices assembled with Control, TD and MTD perovskite films.

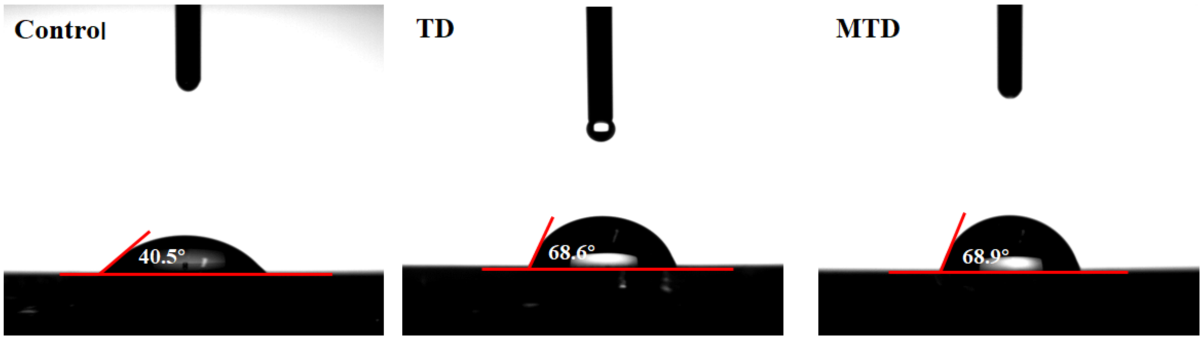

Figure S29. Water contact angles of Control, TD, MTD perovskite films.

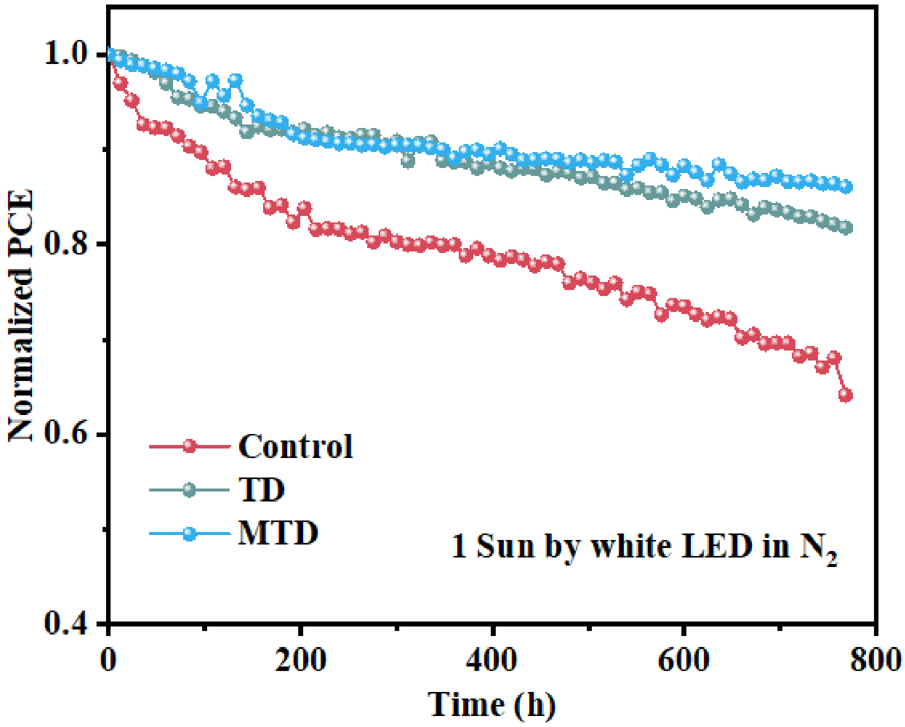

Figure S30. The operational stability test of the unencapsulated PSCs under MPP tracking and continuous light irradiation with a white LED lamp (100 mWcm^{-2}) in a nitrogen environment.

5. Tables

Table S1. Fitting parameters for the TRPL spectra of perovskite films.

Samples	τ_1 (ns)	A_1 (%)	τ_2 (ns)	A_2 (%)	τ_{avg} (ns) *
Control	323.3	9.15	11.59	90.85	901.7
TD	384.2	7.63	1596.6	92.37	1504.1
MTD	767.3	11.59	2029.4	88.41	1883.1

Table S2. Fitting parameters for EIS impedance.

Device	R_s (Ω)	R_{tr} (kΩ)	R_{rec} (kΩ)
Control	49.37	3.880	17.348
TD	48.19	2.683	24.775
MTD	37.53	1.528	2.7995

Table S3. Photovoltaic parameters of champion PSCs.

Sample	Scan direction	V_{oc} (V)	J_{sc} (mA cm^{-2})	*FF* (%)	*PCE* (%)	*HI**
Control	Forward	1.07	24.44	75.34	19.70	0.026
	Reverse	1.08	24.64	76.06	20.24	
TD	Forward	1.12	25.21	76.77	21.87	0.018
	Reverse	1.13	25.24	78.02	22.26	
MTD	Forward	1.15	25.68	81.06	24.15	0.002
	Reverse	1.16	25.68	81.20	24.19	

* denoted the hysteresis index (HI), which is expressed as $HI=(PCE_{reverse}\text{-}PCE_{forward})/PCE_{reverse}$ [8]

6. References

[1] R. Zhao, P. Wang, L. Wang, Y. Zhao, C. Ge, L. Sun, L. Xie, Y. Hua, *Adv. Funct. Mater.* **2024**, *34*, 2307559.

[2] Z. Xiong, S. Chen, P. Zhao, Y. Cho, G. O. Odunmbaku, Y. Zheng, D. J. Jones, C. Yang, and K. Sun, *Sol. RRL* **2021**, *5*, 2100448.

[3] G. Kresse, J. Furthmüller, *Comput. Mater. Sci.* **1996**, *6*, 15.

[4] G. Kresse, J. Furthmüller, *Phys. Rev. B* **1996**, *54*, 11169.

[5] J. P. Perdew, K. Burke, *M. Ernzerhof, Phys. Rev. Lett.* **1996**, *77*, 3865.

[6] G. Kresse, D. Joubert, *Phys. Rev. B* **1999**, *59*, 1758.

[7] P. E. Blöchl, *Phys. Rev. B* **1994**, *50*, 17953.

[8] P. Liu, W. Wang, S. Liu, H. Yang, Z. Shao, *Adv. Energy Mater.* **2019**, *9*, 1803017.
